# A Cancer Exercise Toolkit Developed Using Co-Design: Mixed Methods Study

**DOI:** 10.2196/34903

**Published:** 2022-04-21

**Authors:** Amy M Dennett, Clarice Y Tang, April Chiu, Christian Osadnik, Catherine L Granger, Nicholas F Taylor, Kristin L Campbell, Christian Barton

**Affiliations:** 1 La Trobe Sport and Exercise Medicine Research Centre School of Allied Health, Human Services and Sport La Trobe University Bundoora Australia; 2 Allied Health Clinical Research Office Eastern Health Box Hill Australia; 3 School of Health Sciences Western Sydney University Campbelltown Australia; 4 Department of Physiotherapy School of Primary and Allied Health Care Monash University Frankston Australia; 5 Department of Physiotherapy The University of Melbourne Parkville Australia; 6 Department of Physiotherapy The Royal Melbourne Hospital Parkville Australia; 7 Department of Physical Therapy Faculty of Medicine University of British Columbia Vancouver, BC Canada

**Keywords:** cancer, website, online learning, professional development, physiotherapy, exercise, cancer survivorship, cancer survivor, digital health, online health, online toolkit

## Abstract

**Background:**

Access to exercise therapy for cancer survivors is poor. Professional development to support exercise professionals in delivering these interventions is needed. Few online resources exist for exercise professionals to address this issue.

**Objective:**

To develop and evaluate a freely available online toolkit to support exercise professionals working with cancer survivors.

**Methods:**

A 2-phase, experience-based co-design approach was used to develop and evaluate the online toolkit. The two phases were as follows: 1) needs identification and co-design of resources and platform and 2) pilot evaluation. Four co-design workshops were conducted, transcribed, and thematically analyzed to identify key elements for the toolkit. For the pilot evaluation, a customized survey (the Determinants of Implementation Behavior Questionnaire) was distributed to exercise professionals at baseline and 3 months after launch of the online toolkit to determine its usability, utility, and effectiveness in improving their knowledge, confidence, and behavior. Results were reported as the median and interquartile range and changes were calculated using non-parametric tests. Website analytics described site usage after the initial evaluation.

**Results:**

Twenty-five exercise professionals participated in co-designing 8 key elements of the online Cancer Exercise Toolkit: the homepage and pages for getting started, screening and safety, assessment, exercise prescription, education, locations, and resources. For the pilot evaluation, 277/320 respondents (87% of whom were physiotherapists) from 26 countries completed the survey at baseline, with 58 exercise professionals completing follow-up surveys at 3 months. Exercise professionals’ knowledge, skills, and confidence in delivering exercise therapy to cancer survivors increased 3 months after baseline (items 1, 6, and 8: median score 5, IQR 3 to 6) to follow-up (items 1 and 6: median score 6, IQR 5 to 6; item 8: median score 5, IQR 5 to 7; *P*<.001) on a 1 to 7 Likert scale. Most participants (35/44, 80%) agreed or strongly agreed they would recommend the toolkit to colleagues. In the 6 months following the pilot evaluation, the toolkit received an average of 866 views per month.

**Conclusions:**

The co-designed online Cancer Exercise Toolkit was a useful resource for exercise professionals that may increase their knowledge, skills, and confidence in providing exercise therapy to cancer survivors.

## Introduction

International guidelines support the integration of exercise into cancer care to improve cancer outcomes [1,2]. Well-established evidence shows exercise therapy can reduce cancer-related impairments such as fatigue and improve the health-related quality of life of cancer survivors [1]. Exercise may prevent development of chronic disease, prolong survival, and prevent cancer recurrence in some cancer cohorts, such as breast, colorectal, and prostate cancer [3,4]. Despite compelling evidence that exercise is important for cancer survivors, access to specialized exercise therapy programs for people with cancer is poor, with just 1 in 200 cancer survivors able to participate in an exercise-based rehabilitation program in Australia [5,6].

Skilled exercise professionals are critical for the implementation and delivery of exercise therapy to cancer survivors [7]. Exercise professionals, including physiotherapists and exercise physiologists, are well placed to provide exercise therapy given their expertise in prescribing exercise and behavior change for people with chronic health conditions [8,9]. In Australia alone, there are over 40,000 registered exercise professionals who could provide services to people with cancer [10,11]. Despite their professional training, recent surveys of Australian and Irish physiotherapists found they lack confidence in providing care, including exercise therapy, to cancer survivors [12,13]. Education and practical support are required for exercise professionals to safely and effectively prescribe exercise and monitor progress according to current cancer guidelines [1].

Exercise professionals may be able to develop and consolidate their knowledge through attendance of in-person courses and lectures and passive text-based resources. However, these knowledge sources may be less effective at improving knowledge and skills than active approaches such as e-learning, which provide greater flexibility to cater for individual learning needs [14]. Online material has been shown to be feasible for educating clinicians about exercise, with multimedia innovations, such as video, infographics, quizzes, and podcasts, enhancing clinician engagement [15]. For example, the online Pulmonary Rehabilitation Toolkit [16], developed in Australia over 10 years ago, is now considered an essential reference for physiotherapists and students working in pulmonary rehabilitation [17]. Currently, few similar resources exist to facilitate professional development for exercise professionals working in cancer rehabilitation. With a rapid rise in exercise and cancer research [18,19], it can be challenging for clinicians to keep up with best practices. Online resources may overcome time and cost barriers to professional development and offer convenience for time-poor clinicians [20].

The primary aim of this study is to develop an online toolkit, based on experience-based co-design [21] methods, to provide support to exercise professionals by delivering evidence-based exercise interventions to cancer survivors. A secondary aim is to evaluate the initial use of the online toolkit and explore its effect on exercise professionals’ knowledge, confidence, and behavior.

## Methods

### Study Design

**Figure 1:**
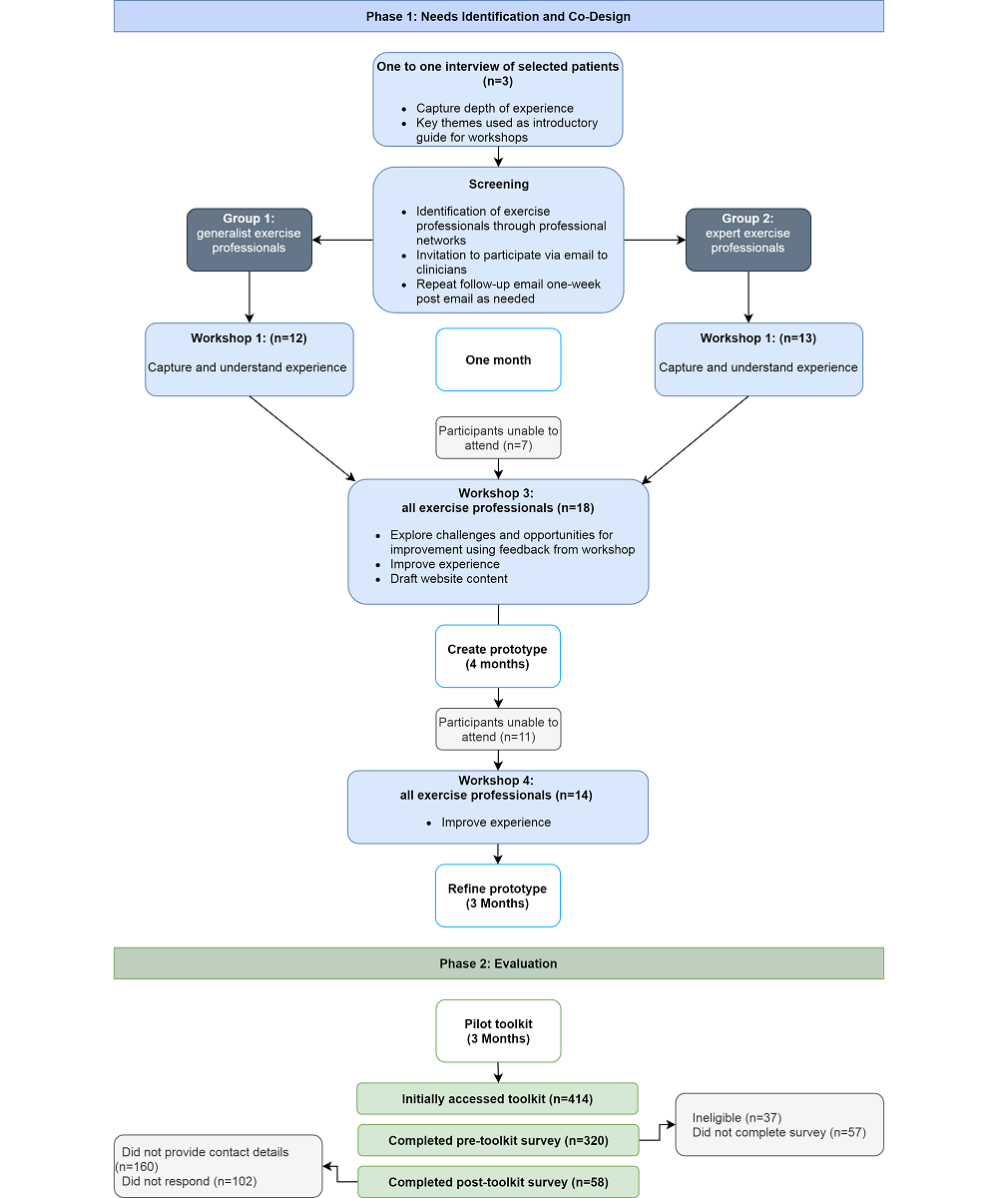
Participant recruitment procedure for the creation and evaluation of the Cancer Exercise Toolkit.

An online toolkit called the Cancer Exercise Toolkit was developed with an experience-based co-design approach [21] using mixed methods between May 2020 and October 2021. Qualitative interviews, workshops, and online surveys informed the toolkit development. The study procedure (Multimedia Appendix 1) was based on the experience-based co-design (EBCD) toolkit [21] and a published study using EBCD to develop a cancer prehabilitation program [22]. EBCD is a collaborative approach to service improvement completed in partnership with end users [21]. Co-design helps researchers build meaningful relationships with research participants [23], whereby users are recognized as experts in their own experiences [24]. The study was completed in two phases: (1) needs identification and co-design of resources for the online platform and (2) pilot evaluation (Figure 1).

### Participants

Two groups of participants were included in the co-design workshops for toolkit development. Group 1 included “generalist” exercise professionals, defined as physiotherapists and exercise physiologists working in other areas who may have occasional contact with cancer survivors. Group 2 included “expert” or experienced cancer exercise professionals, defined as physiotherapists and exercise physiologists who had worked specifically in cancer for at least 2 years. The workshops did not include patients, as exercise professionals were intended to be the end users of this resource. However, patients who had been diagnosed with cancer and participated in exercise-based cancer rehabilitation were invited to participate in a brief video shown to clinicians in the co-design workshops, setting the scene and direction for the session. Snowball sampling was undertaken to recruit participants over a 2-week period. Exercise professionals were invited to participate in the study through an invitation email distributed by a health service and through local professional networks (eg, the Australian Physiotherapy Association). For workshops 1 and 2, it was estimated that 8 to 10 participants in each group would be sufficient to provide varied experiences and contribute to new knowledge [21].

For the pilot evaluation phase of the toolkit, a third group of exercise professionals was recruited. We aimed to recruit a convenience sample of at least 100 exercise professionals over a 3-month period. This sample size assumed that 50% of participants would be confident enough to prescribe exercise therapy to cancer survivors and that this would be sufficient for estimating the expected proportion of sufficiently confident participants with 10% absolute precision and 95% CI [25]. Recruitment was not capped, as participants received recognition for continuing professional development as part of participation.

### Procedure

#### Phase 1: Needs Identification and Co-Design

One-hour semi-structured interviews (Multimedia Appendix 2) were completed via teleconference (Zoom Video Communications) with 3 patients who had participated in cancer rehabilitation in a public subacute hospital in Australia. Interviews were conducted by a member of the research team who had previous experience in conducting qualitative interviews and did not have any prior involvement in the treatment of these patients. The interviews included questions exploring the patients’ journey in participating in an exercise-based cancer rehabilitation program. The videos were independently analyzed by 2 research team members (AD and CT) using an inductive approach to identify key touchpoints of the overall cancer rehabilitation experience [21]. The videos were edited into a short video clip and used at the start of workshops 1 and 2 to set the scene for the sessions.

Separate workshops (workshops 1 and 2, each 1 hour long) with the generalist and expert exercise professionals were conducted to explore areas for health care improvement and identify therapist learning needs. Learning needs identified from the workshop formed the content outline of the new online toolkit. A combined workshop (workshop 3; 1.5-2 hours long) was then held with all the participating exercise professionals to design key content elements and the overall layout of the online toolkit. A prototype online toolkit was developed based on findings from the combined workshop and key cancer rehabilitation literature [1,26,27]. A weblink was sent to exercise professionals attending the workshops to trial the toolkit for 1 month.

Following 1 month of access to the prototype, a second joint workshop (workshop 4; 1.5 hours long) was conducted to facilitate feedback. In this workshop, participant perceptions regarding the strengths and limitations of the new resource were explored. Further refinements to the toolkit were made by the research team following this workshop before it was formally evaluated by the broader exercise community (Phase 2).

Workshops were facilitated by a researcher with experience in EBCD (CT). Two members of the study team (AC and AD) generated field notes to assist in triangulation and data trustworthiness. Project team members acted as observers and additional facilitators for the larger joint workshops. Immediately after each workshop, project team members debriefed with the workshop facilitator and discussed their reflections.

Recordings from all workshops were transcribed, stored, and managed using Microsoft Word and NVivo (version 12). Transcripts were coded independently by 2 reviewers (AD and CT), who used an inductive thematic analysis approach to identify touchpoints from the workshops [28]. The team then came together to discuss and reach consensus on the key touchpoints, which informed the structure and design of the online toolkit. All but 1 team member had experience in conducting qualitative research (Multimedia Appendix 3).

#### Phase 2: Pilot Evaluation

The online toolkit was formally piloted and evaluated with a broader, international sample of exercise professionals, including co-design participants (February 2021 to April 2021). An open online survey, Research Electronic Data Capture (RedCap) [29], was distributed to a large health service and via local professional networks (eg, the Australian Physiotherapy Association and Exercise and Sports Science Australia), as well as international ones (eg, the Canadian Physiotherapy Association and the University of British Columbia Clinical Exercise Physiology Lab) through email and social media pages. Participants gained access to the website after completion of the survey. The survey was completed twice: (1) prior to accessing the website (T0) and (2) 3 months after initially gaining access to the website (T1). The T1 surveys were sent only to participants who provided contact details at the end of the T0 survey. Reminder emails were sent at 7 and 14 days after distribution of the T1 survey. A free professional development event held via webinar was also conducted at follow-up to promote survey completion.

This anonymous online survey (Multimedia Appendix 4) aimed to explore the website’s effectiveness in addressing knowledge gaps, confidence, and behavior in prescribing exercise according to guidelines [1] along with the usability and utility of the toolkit [16]. It comprised 3 sections and took approximately 10 minutes to complete. Section 1 included demographic data. Section 2 included questions derived from the Determinants of Implementation Behavior Questionnaire (DIBQ), which is based on the theoretical domains framework [30]. Domains in the DIBQ show high discriminant validity, reliability, and internal consistency [30]. The 45-item instrument assessed the impact of continuing professional development activities on health professionals’ knowledge, confidence, and implementation behaviors. Each item was measured on a 7-point Likert scale (ranging from 1, “strongly disagree” to 7, “strongly agree”). Item 45 was reverse scaled. Section 3 related to the usability and utility of the website [31] and was included in the follow-up survey only. This survey was tested by members of the research team (AD, CB, and CO) for readability and functionality prior to its distribution. A short quiz created by the researchers was also embedded as a learning tool within the toolkit to test user knowledge related to published recommendations on exercise and cancer [1,26]. Website views at the end of the 3-month trial period (May to October 2021) were reported to identify engagement with the website.

### Data analysis

Survey and website metadata were described using proportions, medians, and interquartile ranges. Content analysis was conducted on open-ended survey questions by 2 researchers (AC and CO) independently. Following recommendations for the analysis of anonymous survey data that cannot be paired [32], differences in DIBQ scores between baseline and follow-up were analyzed using the Mann Whitney *U* test with Bonferroni adjustment for multiple comparisons. A sensitivity analysis was applied to account for dependence in the follow-up survey. This involved using the same sample size at baseline and follow up in a random sample of data from the baseline survey [32]. Data were analyzed using SPSS version 27 (IBM Corp).

### Ethics Approval

This study was reported in accordance with the Consolidated Criteria for Reporting Qualitative Studies [33] and Good Reporting of A Mixed Methods Study [34] checklists and approved by the hospital and university ethics committees (LR 20-020). Workshop participants provided written informed consent. Consent for the online surveys was implied by survey completion.

## Results

### Phase 1: Needs Identification and Co-Design

Twenty-five exercise professionals (13 experts and 12 generalists) participated in the co-design workshops. The co-design group included 21 physiotherapists and 4 exercise physiologists. Thirteen co-design participants worked in hospital settings in Australia. On average, the exercise professionals had 15 years of total experience (Table 1).

**Table 1:** Characteristics of co-design participants.

Characteristics, n (%)			All (N=25)		Expert (n=13)		Generalist (n=12)
**Profession**							
	Physiotherapist	21 (84)		11 (85)		10 (83)	
	Exercise physiologist	4 (16)		2 (15)		2 (17)	
**Setting**							
	Hospital	12 (48)		10 (77)		3 (25)	
	Community	9 (36)		1 (8)		8 (67)	
	Both	1 (4)		1 (8)		0 (0)	
**Funding**							
	Public	14 (56)		8 (62)		7 (58)	
	Private	2 (8)		0 (0)		2 (17)	
	Both public and private	6 (25)		3 (23)		3 (23)	
Years of total experience, mean (SD)			14.8 (8.6)		17.2 (8.0)		12.3 (8.6)

Workshops 1 and 2 identified 5 key touchpoints describing successful cancer rehabilitation programs (Table 2). These touchpoints highlighted the knowledge exercise professionals required to be included in the toolkit for implementation in cancer rehabilitation programs. Overall, touchpoints were similar between the expert and generalist exercise professionals.

Need easy access to latest guidelines for general knowledge...often difficult to keep up to date...

Expert group participant

[Need] access [to] article(s), training... [to be] more confident to safely advocate...to other health professionals.

Generalist group participant

When compared to the generalist group, the experts identified more nuanced, disease-specific knowledge, such as the need for strict infection control procedures and cancer-specific assessments. The importance of practical considerations, understanding the impact of cancer treatment and side effects, and education provision and access were common themes forming the foundational content of the toolkit prototype. These touchpoints informed 8 key sections of the toolkit: the homepage; getting started; screening and safety; assessment; exercise prescription; education; locations; and resources (Multimedia Appendix 5).

In the joint workshop (workshop 3), the exercise professionals agreed the toolkit needed to be simple, practical, and not duplicate existing resources. Participants provided suggestions for existing resources that could be linked or embedded in the toolkit and described a need for templates that could be used in their clinical practice. Website monitoring and updating were identified as critical for the website’s sustained success. At the conclusion of this workshop, the research team drafted the toolkit content. Freely available multimedia resources (videos, infographics, patient handouts, and podcasts) were sourced to supplement information provided on the website rather than creating new multimedia content.

**Table 2:** Key touchpoints from workshops 1 and 2.

Elements of cancer rehabilitation	Common themes	Expert only	Generalist only
Getting started	Setting up the environment, including social support, space, equipment, and group dynamics; communicating with patients how to get started with cancer rehabilitation	Importance of infection control due to work with immunocompromised patients	Whether to deliver therapy one-to-one or in groups; uncertainty as to how to integrate cancer patients with other disease populations; standardized templates and letters
Screening and safety; assessment	Understanding impact of cancer treatment; precautions and contraindications	Discussion of impairment, performance, and quality of life measures used for assessment, including cancer-specific measures	Emphasis on importance and challenges of goal setting
Exercise prescription	Individualization; modification and progression/regression; monitoring fatigue	More emphasis on guidelines and optimal dosage	Patient-centered approach to tailor exercise based on needs and symptoms
Education	Requirement for multidisciplinary input, including psychological and nutritional support and fatigue management; need for resources for both patients and clinicians; inclusion of patient testimonials	N/A^a^	N/A
Access	Poor access to cancer rehabilitation	Acknowledgement of lack of sufficient suitable programs	Difficulty of generating and managing referrals; low confidence of other health professionals to refer patients to cancer rehabilitation

^a^N/A: Not applicable. There were no differences in the themes related to education between the 2 groups.

At the second joint workshop (workshop 4), further refinements were made (Multimedia Appendix 6) including changing the website name to the Cancer Exercise Toolkit [35] and creating a logo. The main feedback was related to navigation and the addition of content. More content was added on special cancer populations, including exercise modifications for specific cancers and side-effects of treatment. The final website was, and still is, a freely available web-based resource that can be self-navigated by users. At the time of evaluation, it comprised 8 sections including relevant information related to implementing exercise-based rehabilitation for cancer survivors (Multimedia Appendix 5 and Multimedia Appendix 7).

### Phase 2: Pilot Evaluation

**Figure 2:**
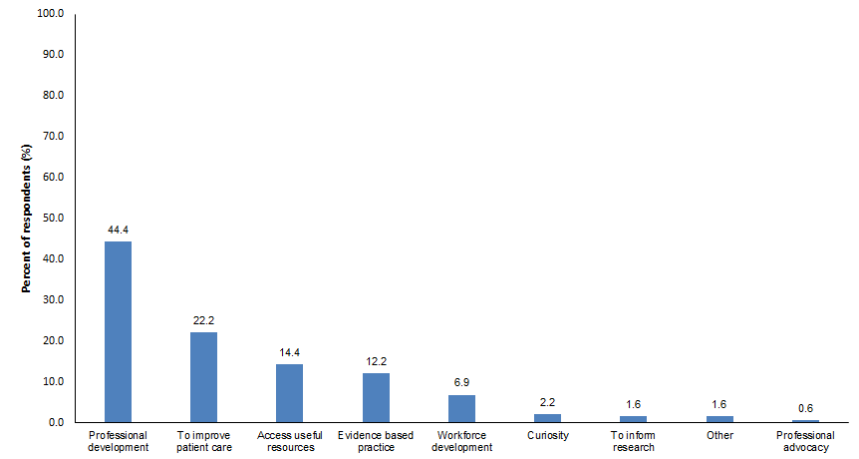
Self-reported motivations for accessing the Cancer Exercise Toolkit website (multiple answers possible).

The website [31] was launched on World Cancer Day (February 4, 2021) and the baseline survey was accessed by 414 people, 37 of whom did not identify as exercise professionals; the survey was terminated. An additional 57 participants did not complete the survey. Respondents who were exercise professionals included 320 clinicians from 26 countries, with most having 5 years or less of cancer-specific experience (Table 3). The majority were physiotherapists (277/320, 87%). Just 120 of the 320 clinicians (38%) worked exclusively in cancer, palliative care, or lymphedema care. The main motivations for accessing the website were for professional development (142/320, 44%) and to improve patient care (17/320, 22%) (Figure 2).

Contact details for follow-up surveys were provided by 160 respondents, of whom 58 completed the follow-up survey (for a response rate of 36%). There were no differences in demographics between those who completed the baseline and follow-up surveys (Table 3).

**Table 3:** Participant characteristics at baseline and in a 3-month follow-up survey.

Characteristics			Baseline (N=320)		3-month follow-up (n=58)
**Discipline, n (%)**					
	Physiotherapy	277 (87)		51 (88)	
	Exercise physiology	43 (13)		7 (12)	
**Country or region, n (%)**					
	Australia	249 (78)		50 (86)	
	Europe/United Kingdom	38 (12)		3 (5)	
	Americas	15 (5)		3 (5)	
	Asia/Pacific	8 (3)		0 (0)	
	Africa	7 (2)		2 (3)	
	Middle East	1 (0.3)		0 (0)	
City-based, n (%)			228 (71)		41 (71)
**Setting, n (%)**					
	Public	159 (50)		29 (50)	
	Private	116 (36)		21 (36)	
	Both public and private	36 (11)		7 (12)	
	Other	9 (3)		0 (0)	
Years of experience, mean (SD)			14 (10)		15 (10)
**Years of cancer-specific experience (if applicable), n (%)**					
	<1	82 (26)		9 (16)	
	1-2	60 (19)		12 (21)	
	3-5	60 (19)		12 (21)	
	6-10	29 (9)		9 (15)	
	>10	25 (8)		7 (12)	
**Primary area of clinical practice, n (%)**					
	Cancer/palliative care/lymphedema	118 (37)		23 (40)	
	Other	200 (63)		35 (60)	
**Proportion of caseload cancer, n (%)**					
	76-100%	61 (19)		14 (24)	
	51-75%	26 (8)		5 (9)	
	26-50%	55 (17)		14 (24)	
	≤25%	174 (54)		25 (43)	
**Highest level of qualification, n (%)**					
	Undergraduate degree	138 (43)		24 (41)	
	Post-graduate certificate	71 (22)		12 (21)	
	Masters by coursework	73 (23)		17 (29)	
	Masters by research	13 (4)		1 (2)	
	PhD	20 (6)		4 (7)	
**Cancer-specific professional development completed^a^**					
	Informal training	175 (55)		0 (0)	
	External courses	173 (54)		0 (0)	
	Post-graduate education	42 (13)		0 (0)	
	Other	23 (7)		0 (0)	
	None	45 (14)		0 (0)	

^a^No responses were gained at follow-up as this question was not asked at follow-up.

### Usage, Usability, and Utility

After the 3-month pilot period, the toolkit received on average 866 views per month. Toolkit usage peaked in June 2021 at 1205 views and declined to 731 views in October 2021.

The most viewed pages were “Locations,” “Patient Education,” and “Precautions and Contraindications” (Multimedia Appendix 8).

Participants found the website useful, easy to understand, and easy to use (items 1 to 4: median score 6, IQR 5-7) (Table 4). Most participants (35/44, 80%) agreed or strongly agreed that they would recommend the Cancer Exercise Toolkit to colleagues. Open-ended feedback received from 11 participants was positive; the following are representative quotes:

Great source, filling a gap; like the pulmonary rehab toolkit.

I had difficulties accessing the toolkit and never got around to sorting out the issue.

Participants suggested some minor improvements to the website relating to accessibility (n=3), website function (n=2), increasing website scope (n=2), and dissemination (n=2).

**Table 4:** Website usability and utility.

Question	Median rating, IQR^a^	Rating 6 or 7 (“strongly agree”), n (%)
Overall, the Oncology Rehabilitation Toolkit website was easy to use (n=44)	6, 5-7	30 (68)
The content of the Oncology Rehabilitation Toolkit website met my expectations (n=44)	6, 5-7	31 (70)
Overall, it was easy to understand the organization of the Oncology Rehabilitation Toolkit website screens, especially the menu levels and the flow of the screens (n=42)	6, 5-7	28 (67)
How useful do you find the Oncology Rehabilitation Toolkit website to be? (n=44)	6, 5-7	29 (66)
I would recommend the Oncology Rehabilitation Toolkit website to my colleagues (n=44)	7, 6-7	35 (80)

^a^Numbers are Likert scales ranging from 1 (“strongly disagree”) to 7 (“strongly agree”)

### Determinants of Implementation Behavior Questionnaire

At baseline, participants rated themselves highest on items relating to their capability to deliver exercise rehabilitation according to guidelines and lowest on items relating to their training and ability to practice delivering exercise rehabilitation (Table 5, Multimedia Appendix 9).

**Figure 3:**
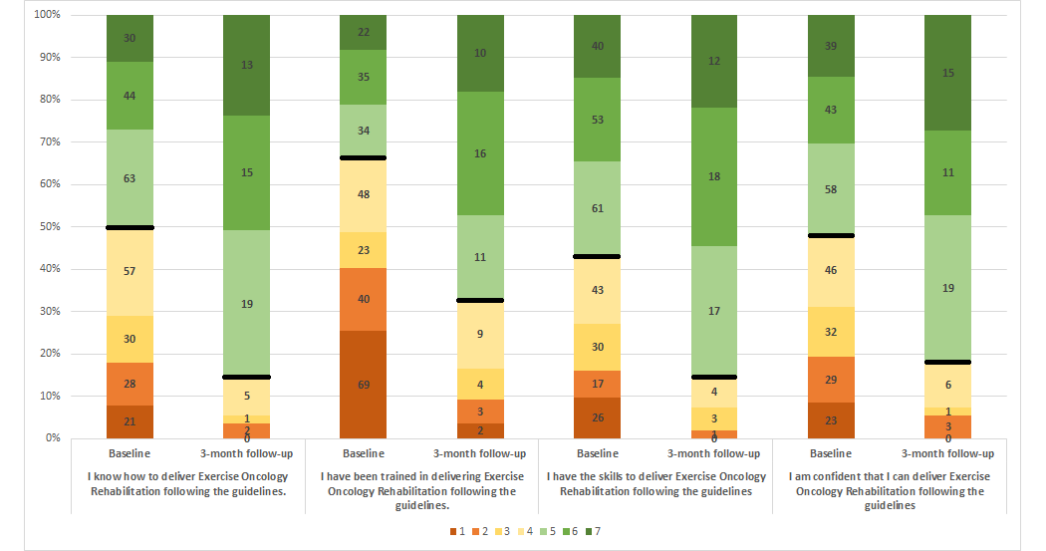
Differences in Determinants of Implementation Behavior (DIBQ) scores between baseline and 3-month follow-up. Figure legend: Shaded data refer to Likert scales ranging from 1 ("strongly disagree") to 7 ("strongly agree"), numbers refer to absolute number of participants who answered survey question.

At the 3-month follow-up, participants self-reported significantly higher scores on items related to knowledge and skills (items 1-7, *P*<.001) and confidence to deliver exercise therapy according to guidelines (items 8 and 9, *P*<.001) (Figure 3, Table 5).

**Table 5:** Determinants of Implementation Behavior Questionnaire. The significance level was set at *P*<.001 (Bonferroni adjustment). Italics indicate significance after the sensitivity analysis was applied. A total of 47 subjects did not complete this section of the survey at baseline. At follow-up, an additional 3 responses were excluded as participants indicated they never accessed the website.

Question		Baseline (N=273)	Follow-up (n=55)	Between-group difference (*P* value)
**Knowledge, median (IQR)**				
	I know how to deliver Exercise Oncology Rehabilitation following the guidelines.	5 (3-6)	6 (5-6)	*<.001*
	Objectives of Exercise Oncology Rehabilitation and my role in this are clearly defined for me.	4 (3-6)	5 (5-6)	*<.001*
	With regard to Exercise Oncology Rehabilitation, I know what my responsibilities are.	5 (3-6)	6 (5-6)	*<.001*
	In my work with Exercise Oncology Rehabilitation, I know exactly what is expected from me.	4 (3-5)	6 (5-6)	*<.001*
**Skills, median (IQR)**				
	I have been trained in delivering Exercise Oncology Rehabilitation following the guidelines.	4 (1-5)	6 (4-6)	*<.001*
	I have the skills to deliver Exercise Oncology Rehabilitation following the guidelines.	5 (3-6)	6 (5-6)	*<.001*
	I am practiced to deliver Exercise Oncology Rehabilitation following the guidelines.	4 (2-5)	6 (4-6)	*<.001*
**Confidence, median (IQR)**				
	I am confident that I can deliver Exercise Oncology Rehabilitation following the guidelines.	5 (3-6)	5 (5-7)	*<.001*
	I am confident that I can deliver Exercise Oncology Rehabilitation following the guidelines even when other professionals with whom I deliver Exercise Oncology Rehabilitation do not do this.	4 (3-6)	5 (5-6)	*<.001*
	I am confident that I can deliver Exercise Oncology Rehabilitation following the guidelines even when there is little time.	4 (3-5)	5 (4-6)	<.001
	I am confident that I can deliver Exercise Oncology Rehabilitation following the guidelines even when participants are not motivated.	4 (3-5)	5 (4-6)	*<.001*

### Triangulation of Data

Qualitative data obtained from clinician workshops converged with quantitative survey data. Participants expressed a need to access information related to published exercise guidelines and described information related to exercise screening and safety as a priority. Areas of traffic on the toolkit were highest for pages related to safety and education (Multimedia Appendix 8). This aligned with survey item scores related to guidelines (“I know how to deliver Exercise Oncology Rehabilitation following the guidelines”; “I have been trained in delivering Exercise Oncology Rehabilitation following the guidelines”; “I have the skills to deliver Exercise Oncology Rehabilitation following the guidelines”; “I am confident that I can deliver Exercise Oncology Rehabilitation following the guidelines”) improving at follow-up.

## Discussion

### Principal Findings

This study identified key learning needs of exercise professionals related to cancer care and facilitated development of the co-designed online Cancer Exercise Toolkit. Learning needs included knowledge of practical considerations for starting a cancer rehabilitation program; how to perform assessment, screening, and safety; and how to prescribe exercise, including tailoring and monitoring. Other important elements described by participants were facilitating access to care, clinician and patient education, and inclusion of templates and forms to support practice. The toolkit had international reach and was described as useful and easy to navigate. The pilot evaluation suggests the Cancer Exercise Toolkit may also improve exercise professionals’ knowledge, skills, and confidence to deliver exercise therapy to cancer survivors.

Knowledge, skills, and confidence of exercise professionals to provide exercise therapy according to guidelines were rated higher after access to the Cancer Exercise Toolkit. This finding indicates that online toolkits such as this could be a useful knowledge translation strategy, supporting previous research showing that online platforms can support delivery of evidence-based practice [36]. The areas of highest traffic on the website after initial piloting included sections related to education, safety, and access. This aligns with the learning needs identified in the co-design workshops and with previous research indicating that these are the areas exercise professionals most lack confidence when managing people with cancer [12]. Building workforce capacity through development of high-quality education and broad dissemination is high on the agenda for the “Moving Through Cancer” movement to embed exercise as part of standard care by 2029 [37]. By improving the knowledge and skills of exercise professionals, it is likely to lead to better quality of care for cancer survivors and improve access to specialized cancer rehabilitation programs.

Most toolkit users were exercise professionals who did not specialize in cancer but were motivated to obtain professional development and improve patient care. Initial survey respondents and users indicated that we achieved a global reach, with more than 400 health professionals from 26 countries accessing the toolkit. This reach is important considering that recent national [38] and international guidelines [1] call for increased access and uptake of exercise services for cancer survivors. Highlighting the need for resources like the Cancer Exercise Toolkit, very few exercise professionals registered in Australia have specialist qualifications or training in cancer care. Moreover, many exercise professionals feel underprepared to practice in cancer care after their entry-level training [12]. Many professional bodies have only started developing post-graduate career pathways in cancer care in the past 5 years [39]. This study found that most clinicians receive their professional development through informal training, which may reflect the scarcity of professional development opportunities traditionally available in this area [12]. The Cancer Exercise Toolkit developed in this study provides generalist and specialist clinicians new opportunities to improve their cancer-specific knowledge and skills to meet increasing demand.

The toolkit appeared to meet clinician needs, being described as easy and useful, with most survey respondents agreeing they would recommend it to their colleagues. Characteristics of the toolkit informed by the co-design process reflected effective web design, such as easy navigation; inclusion of images, logos, and multimedia content; optimal organization, including a hierarchical structure; and content utility, determined by sufficiency, relevancy, quality, and motivational power of the information [40]. While there was a high initial uptake of the website, usage decreased over time. It is possible that participants obtained what they needed from the website when they initially accessed it, and that they therefore did not need to continue visiting it. Planning for ongoing promotion of the toolkit and updates with new content may also be required to improve user engagement. Planned strategies for ongoing sustainability include sharing and promotion via social media and seeking endorsement by key professional bodies. Maintenance of the toolkit will be imperative to ensure its ability to disseminate up-to-date exercise and cancer knowledge and meet clinicians’ professional development needs in the future.

### Strengths and Limitations

This is the first study to describe the development of a freely available toolkit to support exercise professionals working with cancer survivors. The co-design approach ensured end user learning needs were met through tailoring the toolkit based on clinician experience [23]. The effectiveness of co-design in health is not well established. However, qualitative reports indicate that participants in the co-design process have a positive experience, and materials derived from co-design projects are more applicable and acceptable to end users [23]. Co-design methods have commonly been used in curriculum design for secondary and tertiary education [41,42], but not for developing professional development toolkits in a health setting. Applicability of the toolkit was optimized by involving exercise professionals from a variety of clinical settings with a broad range of experience. Our broad dissemination approach, including engaging exercise professionals worldwide, also enhanced the generalizability of the end product.

There were limitations to this study. In the evaluation, only one-third of the original exercise professional participants completed the follow-up survey. Despite multiple attempts to improve engagement with the follow-up survey, including reminder emails and hosting a webinar where survey completion was promoted, the follow-up response rate remained low. This low response rate is consistent with other clinician surveys designed to evaluate physiotherapy professional development initiatives [43] and may be due to lack of time or motivation. To improve response rate, clinicians could be provided with further incentives to complete follow-up surveys, such as prizes, accredited professional development points, or certificates of completion. We were also unable to match participant responses due to the anonymous nature of the survey. It is also possible that the follow-up responses we did receive were from participants who were more interested and invested in cancer rehabilitation; these participants may have reported higher scores. However, the demographics of the participants who completed the follow-up survey were similar to the overall cohort. Additionally, a sensitivity analysis to account for the issue of dependence was conducted to increase the confidence in our results. Our inclusion of exercise professionals involved in the co-design of the toolkit during the evaluation phase also could have biased the outcome. However, we included this group to optimize the sample size available for evaluation, and to ensure that the changes made following the workshops were appropriate. Other health professionals, such as occupational therapists, dietitians, nurses, and doctors, were excluded, as they are not traditionally involved with specialist exercise prescription for cancer survivors. The website was developed for the Australian context. Health systems in other parts of the world may differ, and the content may need to be adapted to meet their needs. Despite this, positive feedback was received from participants from other countries, indicating that cross-cultural adaptation would likely be acceptable. Lastly, online resources may not be as effective at improving clinician behavior as more active learning strategies, such as workshops and mentoring [44].

### Conclusion

This study described the development of the co-designed Cancer Exercise Toolkit. The toolkit was accessed by physiotherapists and exercise physiologists who described the website as valuable and easy to use. Exercise professionals rated their knowledge, skills, and confidence higher after accessing the website, indicating that it may be an effective alternative or complement to traditional professional development. The Cancer Exercise Toolkit may help improve access to exercise therapy and improve the effectiveness of care for cancer survivors through greater capability of the exercise professional workforce.

## Appendix

Multimedia Appendix 1 Experience-based co-design (EBCD) steps.

Multimedia Appendix 2 Patient interview schedule.

Multimedia Appendix 3 Backgrounds of the research team.

Multimedia Appendix 4 Evaluation survey.

Multimedia Appendix 5 Description of website content.

Multimedia Appendix 6 Website changes.

Multimedia Appendix 7 Screenshot example of Cancer Exercise Toolkit.

Multimedia Appendix 8 Cancer Exercise Toolkit Visits.

Multimedia Appendix 9 Full Determinants of Implementation Behavior Questionnaire (DIBQ) outcomes.

## Abbreviations

DIBQ: Determinants of Implementation Behavior Questionnaire
EBCD: experience-based co-design
