# Supplementary material for: A Cancer Exercise Toolkit Developed Using Co-Design: Mixed Methods Study
Source: JMIR Cancer. 2022 Apr 21;8(2):e34903. doi: 10.2196/34903 (PMC9073617; doi:10.2196/34903)
Supplement: Multimedia Appendix 1 [file cancer_v8i2e34903_app1.docx]

## Appendix 1 – The methodological steps of EBCD to develop the Cancer Exercise Toolkit

| **Co-design steps** | **Description of work to undertake** |
| --- | --- |
| - **Setting the plan** | Defining the problem, challenge and opportunity as described in the protocol’s background and rationale. In brief,  Problem:   - No resources available to support exercise professionals delivering exercise-based cancer rehabilitation. - Low confidence and knowledge of exercise professionals in delivering aspects of exercise-based cancer rehabilitation.   Challenge:   - To understand the learning needs of exercise professionals who deliver exercise-based cancer rehabilitation and what resources they need to improve their confidence in delivering cancer rehabilitation programs.   Opportunity   - To explore ideas together with patients, and health professionals to develop a comprehensive online cancer rehabilitation resource.   Governance for this project will be provided by a steering committee with expertise in co-design, clinical practice, research, quality improvement, implementation and sustainability of improvements. This steering committee will provide strategic oversight as we develop, implement, evaluate and sustain the Cancer Exercise toolkit. Reporting to this committee will occur through bi-monthly snapshot reports by the operational team. |
| - **Engaging with patients and staff** | People will be invited to participate in co-designing the online resource using existing organisational infrastructure to seek engagement.  People with cancer who are currently participating in cancer rehabilitation or recently completed cancer rehabilitation will be engaged for the following purpose:   - **Selected Interviews for Filming** - Three suitable patients will be identified through enrolments of cancer rehabilitation programs at the health service. - Potential patients who are willing to share their stories and indicate an ongoing commitment to participate in consumer engagement activities, will be flagged by the health professionals who have previously had contact with them during their course of cancer rehabilitation. These patients will need to be willing to provide both positive and negative experiences of their journey. - Patients will be approached via phone by their treating clinician and invited to participate in the study with a specific invitation to be interviewed and filmed to capture their narrative. **PICF A** will be given in person or sent via mail or email. - The semi-structured interview schedule for the patient are provided in **Appendix 2**. This will serve as a guide for the interviewer and a copy will be sent to the interviewee in advance to allow for preparation. A member of the project team who is experienced in conducting semi-structured interviews and who does not have any prior involvement with the patient’s treatment will be conducting the interviews.   *Health professionals -2 groups Generalist and Expert*  For health professionals, initial engagement will be via email to exercise professionals (physiotherapists and exercise physiologists) and occur through established communication channels. An explanation of the concept and **PICF B** will be provided.  Two groups of health professionals will be interviewed: generalists and experienced exercise professionals. We aim to engage approximately 8-10 exercise professionals in each group (up to 20 total) who work in a variety of settings (acute, sub-acute, community health). A broad approach using professional networks will be taken as it is anticipated there may be challenges with scheduling and potential attrition due to exercise professionals other time and work commitments. |
| - **Capture the experience** | The experiences of patients, caregivers and health professionals will be captured in this phase using the following methods:   1. **Selected interviews** will occur with 3 patients as described above to capture depth of experience. To capture their narrative, these interviews will be filmed (using teleconference software) or audio-recorded (if via telephone only) as recommended in the published EBCD toolkit (The King’s Fund 2016). The filmed narratives will be edited and relevant sections used in the subsequent meetings to highlight key moments of truth or ‘touch points’. See **Appendix 2** for patient interview schedule.   To understand their experiences, emotional mapping will be used – See Step 4 below “Understand the experience”   1. **Workshop 1+2 Exercise professionals (up to 60 minutes teleconference)** – a total of 16-20 exercise professionals will be invited. The experienced and generalist groups will be interviewed separately. After initial project explanation and priming, relevant sections of the filmed narratives will serve as an introductory guide to the facilitated discussion. The group will be asked the following broad questions: 2. With regard to the patient journey from time of receiving cancer diagnosis to conclusion of treatment and their perspective of delivering rehabilitation and exercise services, what has been working well? What has not? What ideas do you have to improve this experience as a healthcare provider? 3. If an online toolkit of resources is proposed as a potential solution, how might this occur or what might it look like? What are the barriers, facilitators and pragmatic steps we need to consider to set up an online Cancer Exercise toolkit for physiotherapists and other exercise professionals? 4. What is your most preferred way of learning? Why? 5. What online resources have you found useful in the past?   **Workshop 3 (1.5 to 2 hours teleconference) – ALL Exercise professionals** – Exercise professionals gather again virtually to hear each other’s and the patient’s views and perspectives and move forward to co-design from there. See below “Improve the experience.” |
| - **Understand the experience** | The project team organizes learning and insights in the form of an experience map. This requires linking of emotions (highs and lows) experienced through the survivors’ journey (e.g. confident or scared), to the point in the patient journey where it occurred known as ‘touch points’ (e.g. Receiving the cancer diagnosis). This information can generate the patients’ story and a process map to which emotions are matched. A similar map will be used for the staff perspective and reviewed for similarities or differences. These maps will be used at the events described above. |
| - **Improve the experience** | Building the Cancer Exercise toolkit - exercise professionals will work together during **workshop 3** to build the Cancer Exercise toolkit and create learning modules based on feedback from workshop 1 and 2. This will include the following considerations and identification of solutions:   - 1. What should be included in a toolkit for exercise-based oncology rehabilitation for clinicians? What is most important/least important?   2. What existing patient resources would be included? What other patient resources (if any) would need to be developed as part of the Cancer Exercise Toolkit?   3. How should the website be layed out?   4. Other logistics – E.g. marketing to engage other users (e.g. via social media, hospital, professional networks), and sustainability.   Once the ideal online resource is established based on the perspectives and design from the group, the project team will test the prototype with the exercise professionals who participated in workshops 2 and 3. Exercise professionals from the initial workshops will be invited to **workshop 4** to further refine the online toolkit and any necessary adjustments will be made in preparation for a formal pilot with a broader group of physiotherapists for evaluation.  **Workshop 4 (1.5 hours teleconference) -**  The following considerations and identification of solutions will be discussed:   - 1. What aspects of the content did you find useful/not useful and why?   2. How did you find the following: features, usability, accessibility, navigation?   3. What aspects of the website layout did you find useful/not useful and why?   4. What further recommendations would you make regarding the online toolkit? |
| - **Measure the experience** | Initial patient experience will be acquired using the methods described in “Capture the experience” above.  After workshop 4, the final version of the online toolkit will be piloted with a broader group of physiotherapists. A survey will be distributed at initial access to the toolkit and after 3 months of access to evaluate the impact of the online resource. This survey will include a Determinants of Implementation Behaviour Questionnaire and website usability questionnaire. Upon completion of the three-month pilot of the online toolkit, an hour celebratory event will be held inviting all participants of the project to share the outcomes of the project and to gather further feedback and suggestions. |
