# Supplementary material for: A Cancer Exercise Toolkit Developed Using Co-Design: Mixed Methods Study
Source: JMIR Cancer. 2022 Apr 21;8(2):e34903. doi: 10.2196/34903 (PMC9073617; doi:10.2196/34903)
Supplement: Multimedia Appendix 2 [file cancer_v8i2e34903_app2.docx]

## Appendix 2 - Patient Interview Schedule

**The story of your care experience.**

**Introduction**

Thank you for agreeing to take part in this project. The aim of today’s session is to explore your journey and experiences with the health service from diagnosis to your through to your participation in oncology rehabilitation. The findings of this interview will assist in the development of an online toolkit about exercise-based oncology rehabilitation for exercise professionals working in cancer care.

With your consent, we would like to video record the interview. We may ask you if we can use some of the footage from your interview as part of a short edited film that will be shown to staff and patients with cancer. We will let you look at the film and you can decide if you are happy for us to use it. If you do not wish to have this interview video recorded, that is ok as we anticipate we would still be able to learn from your valuable insights.

**1 Your journey so far…**

**1.1 Tell me about your journey so far (opportunity to talk about what has happened etc)**

- What is your first memory of being diagnosed with cancer?
- Describe how you felt physically and emotionally when diagnosed with cancer. What coping mechanisms did you and your caregiver use at that time?
- What was it like to hear your cancer treatment options? How was the information delivered to you?
- Do you have any comments with regards to the information delivery both from a content and process perspective about medical treatment for your prostate cancer? Can you comment on the timing of the delivery of information?
- How did you make the decision around your treatment? What was the timeframe between your decision and treatment starting? Were there any difficulties with making your decision?
- How did you prepare for treatment?
- What was your experience with treatment?
- Describe your recovery.
  1. **Rehabilitation questions**:
- When did you receive rehabilitation after your cancer diagnosis?
- How did you find out about oncology rehabilitation?
- Describe your experience with the oncology rehabilitation team.
- What was the environment like when attending oncology rehabilitation? Did you feel comfortable in this setting? Why/why not? Would you have any suggestions for improvement?
- What were the benefits from oncology rehabilitation?
- Were there any barriers to attending oncology rehabilitation?
- Outline any difficulties you may have experienced when interacting with the staff and health service (i.e. making contact or arranging appointments)

**2.1 Overall satisfaction**

- Broadly speaking, how satisfied have you been so far with your cancer care experience (both oncology rehabilitation and non-rehabilitation related)?

**2.2 The information you received**

- What type of information (in terms of content) was provided throughout your cancer journey apart from the medical treatment of cancer? What was your experience with this?
- What type of information about exercise and/or rehabilitation was given to you throughout your cancer journey?
- What resources did you find most useful?
- Was there any further information that you would have liked to receive about exercise and rehabilitation prior to commencement of treatment?
- Have there been times when you have been given conflicting or contradictory information about exercise and rehabilitation? Can you describe those instances?
- Reflecting back on the way the information was delivered, could you provide further suggestions to improve the delivery of information?

**2.3 Information health professionals should know**

- What information about exercise do you think is most important that your physiotherapist should know?
- What resources should your physiotherapist have access to to improve your care?
- What information (if any) was your physiotherapist unsure about when having discussion with you about exercise?
- How could your physiotherapist best support you to continue exercising after you have finished oncology rehabilitation?

**2.4 How much influence you had**

- Would you have liked to have been more or less involved in your care?

**3 Best and worst bits…**

**3.1** What were the best and worst parts of your cancer rehabilitation experience?

**3.2** Are there important points that you think we should focus on to improve the experience of people attending oncology rehabilitation?

**THANK YOU**
