# Supplementary material for: A Cancer Exercise Toolkit Developed Using Co-Design: Mixed Methods Study
Source: JMIR Cancer. 2022 Apr 21;8(2):e34903. doi: 10.2196/34903 (PMC9073617; doi:10.2196/34903)
Supplement: Multimedia Appendix 3 [file cancer_v8i2e34903_app3.docx]

Appendix 3: Backgrounds of research team members

For reflexivity, backgrounds of the researchers are presented. The principal researcher (AD) is a physiotherapist employed in a cancer rehabilitation program with experience in conducting qualitative research. The lead facilitator (CT) is a physiotherapist and researcher with experience in qualitative and co-design research methods. AC is a physiotherapist working in a cancer rehabilitation program. CB, CO, CG, NT and KC are physiotherapists working in a university with research experience in exercise for various chronic diseases and qualitative research. Some participants in the co-design workshops were known to the research team through professional networks.
