# Supplementary material for: A Cancer Exercise Toolkit Developed Using Co-Design: Mixed Methods Study
Source: JMIR Cancer. 2022 Apr 21;8(2):e34903. doi: 10.2196/34903 (PMC9073617; doi:10.2196/34903)
Supplement: Multimedia Appendix 4 [file cancer_v8i2e34903_app4.docx]

**Appendix 4 – Evaluation survey**

**Section 1: About you**

1. Which Country are you from?
   1. Australia
   2. Other (Please describe)
2. Which state or territory do you work in? (if select Australia)
   1. Victoria
   2. New South Wales
   3. Queensland
   4. Northern Territory
   5. Western Australia
   6. South Australia
   7. Tasmania
   8. Australian Capital Territory
3. What region do you work in?
   1. Metropolitan
   2. Regional
   3. Rural
   4. Other:
4. What setting do you work in?
   1. Public
   2. Private
   3. Both Public and Private
   4. Other
5. Are you a:
   1. Physiotherapist
   2. Exercise Physiologist (Exercise & Sport Science

Australia Accredited Exercise Physiologist (ESSA-AEP), Canadian Society for Exercise Physiology Certified Exercise Physiologist (CSEP-CEP), ACSM Certified Clinical Exercise Physiologist (ACSM-CEP) or equivalent)

- 1. Other (please specify) (if this option is selected, the survey will be terminated)

1. How many years have you been working as a qualified physiotherapist or exercise physiologist? (please enter whole number in years)
2. If applicable, how many years have you worked specifically in the area of cancer? (please enter whole number in years) _____________ /Not applicable
3. What is your primary area of clinical practice ?
   1. Cancer/ Palliative Care/Lymphodema
   2. An area other than Cancer/ Palliative Care/Lymphodema (please describe)_____________
4. What proportion of your case load is treating people with cancer?
   1. 76-100%
   2. 51-75%
   3. 26-50%
   4. ≤25%
5. What is your highest level of education?
   1. Undergraduate degree or entry-level qualification
   2. Post-graduate certificate
   3. Masters by coursework
   4. Masters by research
   5. Professional doctorate
   6. PhD
   7. Other______________________
6. What professional development have you received specific to the area of exercise and cancer (Tick all that apply)?
7. None
8. Informal training on the job from other staff (including tutorials and in-services)
9. External workshops or courses
10. Post-graduate education specialising in cancer
11. Please describe______________
12. What are your main motivations for accessing the oncology rehabilitation toolkit?
    1. __________________________________________________________

**Section 2: Determinants of Implementation Behaviour Questionnaire**

The following section involves completion of a 45-item validated instrument called the Determinants of Implementation Behaviour Questionnaire. It is anticipated to take 5 mins to complete. Please answer each of the following questions by indicating the number that best represents your opinion of **exercise-based oncology rehabilitation for people with cancer**. Some questions may seem similar to you, but they measure different aspects of your opinion of exercise prescription for people with cancer. The guidelines referred to in each question are the [American College of Sports Medicine Guidelines for Exercise and people with Cancer 2019](https://www.acsm.org/docs/default-source/files-for-resource-library/exercise-guidelines-cancer-infographic.pdf?sfvrsn=c48d8d86_4).

| 1. I know how to deliver Exercise Oncology Rehabilitation following the guidelines. | Strongly disagree Strongly agree |  |
| --- | --- | --- |
|  | 1 2 3 4 5 6 7 | |
| 1. Objectives of Exercise Oncology Rehabilitation and my role in this are clearly defined for me. | Strongly disagree Strongly agree  1 2 3 4 5 6 7 | |
| 1. With regard to Exercise Oncology Rehabilitation, I know what my responsibilities are. | Strongly disagree Strongly agree |  |
|  | 1 2 3 4 5 6 7 | |
| 1. In my work with Exercise Oncology Rehabilitation, I know exactly what is expected from me. | Strongly disagree Strongly agree |  |
|  | 1 2 3 4 5 6 7 | |
| 1. I have been trained in delivering Exercise Oncology Rehabilitation following the guidelines. | Strongly disagree Strongly agree |  |
|  | 1 2 3 4 5 6 7 | |
| 1. I have the skills to deliver Exercise Oncology Rehabilitation following the guidelines. | Strongly disagree Strongly agree |  |
|  | 1 2 3 4 5 6 7 | |
| 1. I am practiced to deliver Exercise Oncology Rehabilitation following the guidelines. | Strongly disagree Strongly agree |  |
|  | 1 2 3 4 5 6 7 | |
| 1. I am confident that I can deliver Exercise Oncology Rehabilitation following the guidelines. | Strongly disagree Strongly agree |  |
|  | 1 2 3 4 5 6 7 | |
| 1. I am confident that I can deliver Exercise Oncology Rehabilitation following the guidelines even when other professionals with whom I deliver Exercise Oncology Rehabilitation do not do this. | Strongly disagree Strongly agree |  |
|  | 1 2 3 4 5 6 7 | |
| 1. I am confident that I can deliver Exercise Oncology Rehabilitation following the guidelines even when there is little time. | Strongly disagree Strongly agree |  |
|  | 1 2 3 4 5 6 7 | |
| 1. I am confident that I can deliver Exercise Oncology Rehabilitation following the guidelines even when participants are not motivated. | Strongly disagree Strongly agree |  |
|  | 1 2 3 4 5 6 7 | |
| 1. I have control over delivering Exercise Oncology Rehabilitation following the guidelines. | Strongly disagree Strongly agree | |
|  | 1 2 3 4 5 6 7 | |
| 1. For me, delivering Exercise Oncology Rehabilitation following the guidelines is | Very difficult Very easy  1 2 3 4 5 6 7 | |
| 1. For me, performing the intake is (very difficult – very easy). | Very difficult Very easy  1 2 3 4 5 6 7 | |
| 1. For me, delivering the training program is | Very difficult Very easy  1 2 3 4 5 6 7 | |
| 1. For me, performing the evaluation is | Very difficult Very easy  1 2 3 4 5 6 7 | |
| 1. For me, giving attention to participant’s maintenance of PA behavior outside Exercise Oncology Rehabilitation is | Very difficult Very easy  1 2 3 4 5 6 7 | |
| 1. For me, reporting about the Exercise Oncology Rehabilitation to the referring professional is | Very difficult Very easy  1 2 3 4 5 6 7 | |
| 1. For me, delivering Exercise Oncology Rehabilitation following the guidelines is | Not useful at all Very useful  1 2 3 4 5 6 7 | |
| 1. For me, delivering Exercise Oncology Rehabilitation following the guidelines is | Not worthwhile at all Very worthwhile  1 2 3 4 5 6 7 | |
| 1. For me, delivering Exercise Oncology Rehabilitation following the guidelines is | Not pleasurable at all Very pleasurable  1 2 3 4 5 6 7 | |
| 1. For me, delivering Exercise Oncology Rehabilitation following the guidelines is | Not interesting at all Very interesting  1 2 3 4 5 6 7 | |
| 1. If I deliver Exercise Oncology Rehabilitation following the guidelines Exercise Oncology Rehabilitation will be most effective. | Strongly disagree Strongly agree  1 2 3 4 5 6 7 | |
| 1. If I deliver Exercise Oncology Rehabilitation following the guidelines, participants will appreciate this. | Strongly disagree Strongly agree  1 2 3 4 5 6 7 | |
| 1. If I deliver Exercise Oncology Rehabilitation following the guidelines, this will strengthen the collaboration with professionals with whom I deliver Exercise Oncology Rehabilitation. | Strongly disagree Strongly agree  1 2 3 4 5 6 7 | |
| 1. If I deliver Exercise Oncology Rehabilitation following the Guidelines, I will feel satisfied. | Strongly disagree Strongly agree  1 2 3 4 5 6 7 | |
| 1. If I deliver Exercise Oncology Rehabilitation following the Guidelines, it will help participants to be more physically active. | Strongly disagree Strongly agree  1 2 3 4 5 6 7 | |
| 1. It is possible to tailor Exercise Oncology Rehabilitation to participants’ needs? | Strongly disagree Strongly agree  1 2 3 4 5 6 7 | |
| 1. It is possible to tailor Exercise Oncology Rehabilitation to professionals’ needs? | Strongly disagree Strongly agree  1 2 3 4 5 6 7 | |
| 1. Exercise Oncology Rehabilitation costs little time to deliver. | Strongly disagree Strongly agree  1 2 3 4 5 6 7 | |
| 1. Exercise Oncology Rehabilitation is compatible with daily practice | Strongly disagree Strongly agree  1 2 3 4 5 6 7 | |
| 1. Exercise Oncology Rehabilitation is simple to deliver | Strongly disagree Strongly agree  1 2 3 4 5 6 7 | |
| 1. Most people who are important to me think that I should deliver Exercise Oncology Rehabilitation following the guidelines. | Strongly disagree Strongly agree  1 2 3 4 5 6 7 | |
| 1. Professionals with whom I deliver Exercise Oncology Rehabilitation think I should deliver Exercise Oncology Rehabilitation following the guidelines. | Strongly disagree Strongly agree  1 2 3 4 5 6 7 | |
| 1. Professionals with whom I deliver Exercise Oncology Rehabilitation deliver Exercise Oncology Rehabilitation following the guidelines | Strongly disagree Strongly agree  1 2 3 4 5 6 7 | |
| 1. Other professionals who work with Exercise Oncology Rehabilitation deliver Exercise Oncology Rehabilitation following the guidelines | Strongly disagree Strongly agree  1 2 3 4 5 6 7 | |
| 1. I can count on support from professionals with whom I deliver Exercise Oncology Rehabilitation when things get tough around delivering Exercise Oncology Rehabilitation following the guidelines. | Strongly disagree Strongly agree  1 2 3 4 5 6 7 | |
| 1. Professionals with whom I deliver Exercise Oncology Rehabilitation are willing to listen to my problems with delivering Exercise Oncology Rehabilitation following the guidelines. | Strongly disagree Strongly agree  1 2 3 4 5 6 7 | |
| 1. Professionals with whom I deliver Exercise Oncology Rehabilitation are helpful with delivering Exercise Oncology Rehabilitation following the guidelines. | Strongly disagree Strongly agree  1 2 3 4 5 6 7 | |
| 1. Delivering Exercise Oncology Rehabilitation following the guidelines is something I do automatically. | Strongly disagree Strongly agree  1 2 3 4 5 6 7 | |
| 1. Delivering Exercise Oncology Rehabilitation following the guidelines is something I do without having to consciously remember | Strongly disagree Strongly agree  1 2 3 4 5 6 7 | |
| 1. Delivering Exercise Oncology Rehabilitation following the guidelines is something I do without thinking. | Strongly disagree Strongly agree  1 2 3 4 5 6 7 | |
| 1. Delivering Exercise Oncology Rehabilitation following the guidelines is something I start doing before I realize I am doing it | Strongly disagree Strongly agree  1 2 3 4 5 6 7 | |
| 1. Delivering Exercise Oncology Rehabilitation following the guidelines is something I seldom forget. | Strongly disagree Strongly agree  1 2 3 4 5 6 7 | |
| 1. Delivering Exercise Oncology Rehabilitation following the guidelines is something I often forget. | Strongly disagree Strongly agree  1 2 3 4 5 6 7 | |

Thank you for your participation. Please enter your email so we can provide you with access to the website link (Your email will **not** be linked to your survey responses).

Name:

Email:

**Section 3: Usability and utility** (Post-implementation only (Coursaris 2014)):

In this section, please reflect on your use of the Oncology Rehabilitation Toolkit over the past **3 months** and answer the following questions.

| 1. Overall, the Oncology Rehabilitation Toolkit website was easy to use. | Strongly disagree Strongly agree |  |
| --- | --- | --- |
|  | 1 2 3 4 5 6 7 | |
| 1. The content of the Oncology Rehabilitation Toolkit website met my expectations. | Strongly disagree Strongly agree  1 2 3 4 5 6 7 | |
| 1. Overall, it was easy to understand the organization of the Oncology Rehabilitation Toolkit website screens,   especially the menu levels and the flow of the screens. | Strongly disagree Strongly agree |  |
|  | 1 2 3 4 5 6 7 | |
| 1. How useful do you find the Oncology Rehabilitation Toolkit website to be? | Not at all Very Useful |  |
|  | 1 2 3 4 5 6 7 | |
| 1. I would recommend the Oncology Rehabilitation Toolkit website to my colleagues | Strongly disagree Strongly agree |  |
|  | 1 2 3 4 5 6 7 | |

6. What recommendations do you have for improving the accessibility of the site? ___________________________________________________________________

7. Additional comments and/or recommendations for future enhancements: ___________________________________________________________________

**Section 4: Quiz (embedded in website)**

The following questions relate to your knowledge of key issues that may affect decision-making regarding evaluation and prescription of exercise and physical activity for people with cancer. It is presented in three separate sections (screening and precautions; patient assessment; and exercise prescription).

**Screening and precautions**

1. According to the National Comprehensive Cancer Network (United States), medical clearance from a medical professional should be sought for cancer survivors prior to engaging in exercise….
2. Always
3. Sometimes
4. Never
5. A low blood neutrophil count may suggest a patient is immunocompromised. According to Santa Mina et al 2018, what is the recommended neutrophil threshold cut off below which physician clearance is recommended to be sought?
6. 0.5x10^9^/L
7. 1 x 10^9^/L
8. 1.5 x 10^9^/L
9. 2 x 10^9^/L
10. 2.5 x 10^9^/L
11. A low haemoglobin count may suggest a patient is at greater risk of anaemia. According to Santa Mina et al 2018, what is the recommended haemoglobin threshold cut off below which physician clearance is recommended to be sought?
12. 100 g/L
13. 90 g/L
14. 80 g/L
15. 70 g/L
16. 60 g/L
17. People with lymphoedema should not do strength training
18. True
19. False
20. What are common side-effects associated with use of anthracycline chemotherapy (e.g. doxorubicin) (select all that apply)
21. Cardiotoxicity
22. Peripheral neuropathy
23. Muscle wasting
24. Osteoporosis
25. Nausea and vomiting

**Patient assessment**

1. An Australia-modified Karnofsky Performance Score of **60** would indicate the following physical performance status :
2. Requires occasional assistance but is able to care for most of their needs.
3. Normal; no complaints; no evidence of disease
4. Cares for self; unable to carry on normal activity or to do active work
5. Requires considerable assistance and frequent medical care
6. In bed more than 50% of the time
7. Appropriate examples of validated tests of aerobic capacity/ exercise tolerance for people with cancer may include (select all that apply)
8. 10-metre gait speed
9. 6-minute walk test
10. Modified Bruce Treadmill Test
11. 5-times sit to stand
12. Incremental shuttle walk test
13. The measure of cancer-related fatigue with the highest level of evidence according to the EDGE Taskforce is the
14. Profile of Mood States
15. General Fatigue Scale
16. Brief Fatigue Inventory
17. Visual Analogue Scale for Fatigue
18. PROMIS Cancer Fatigue Short Form

**Exercise prescription**

1. According to the American College of Sports Medicine, what is the recommended basic strength training prescription for improving physical function in people with cancer?
2. 40%-65% of 1RM, 15-20 reps, 4 sets
3. 50%-60% of 1RM, 15-20 reps, 2 sets
4. 60%–75% 1-RM, 2 sets 8-12 reps
5. 70%-85% 1RM, 8-12 reps, 2 sets
6. Intensity as tolerated, 8-12 reps, 2 sets
7. How could exercise be modified for someone with asymptomatic bony metastases of the spine (tick all that apply)
8. Avoid high impact load (e.g. jumping)
9. Do functional exercises only
10. Avoid resistance training
11. Avoid hyperflexion, extension and dynamic twisting with resistance
12. Do aerobic training only
13. What advice should be given to patients at risk of upper limb lymphoedema when they undertake exercise to reduce or prevent symptoms? (select all that apply)
14. A compression garment should be worn during exercise at all times
15. Limb girth should be measured before and after all training sessions
16. Compression garments may be worn if patient’s prefer to do so
17. Wearing compression garments during exercise is not required
18. Patients should not do resistance training with the affected upper limb
19. Patients with mild to moderate fatigue should participate in aerobic exercise at an intensity of approximately ___ HR max (select all that apply)
20. 30%
21. 45%
22. 65%
23. 80%
24. Fatigue is a contra-indication to performing in aerobic exercise
25. The American College of Sports Medicine recommends people with cancer participate in regular exercise to improve quality of life. What duration and frequency of exercise training should be completed in order to achieve this?
26. Moderate-intensity aerobic exercise for 30 minutes, 3 times per week and resistance training twice weekly
27. Moderate-intensity aerobic exercise for 30 minutes, 5 times per week and resistance training twice weekly
28. Moderate-intensity aerobic exercise for 20 minutes daily and resistance training twice weekly
29. Moderate-intensity exercise for 30 minutes, 3 times per week only
30. High-intensity aerobic exercise for 10 minutes, daily

Thank you for your participation. Please enter your email so we can provide you with access to the website link (Your email will **not** be linked to your survey responses).

Name:

Email:
