# Supplementary material for: A Cancer Exercise Toolkit Developed Using Co-Design: Mixed Methods Study
Source: JMIR Cancer. 2022 Apr 21;8(2):e34903. doi: 10.2196/34903 (PMC9073617; doi:10.2196/34903)
Supplement: Multimedia Appendix 5 [file cancer_v8i2e34903_app5.docx]

Appendix 5. Description of cancer exercise toolkit and content

| **Section** | **Content** |
| --- | --- |
| Getting Started | Describes practical strategies and tools to start an exercise-based cancer rehabilitation program including information on referrals, equipment and resourcing. |
| Screening and Safety | Describes safety precautions for exercising people with cancer. Also includes information about common cancer treatments, side-effects and cancer populations. |
| Assessment | Provides guidance for subjective and physical assessment of exercise including links to recommended outcome measures. |
| Exercise Prescription | Provides guidance for exercise prescription for people with cancer based on current guidelines. Includes suggestions for exercise modification and progression and case studies. |
| Education | Includes links to existing resources including key publications, videos, infographics and podcast for patients and health professionals. Also includes a section on non-exercise education resources for patients and information about multidisciplinary education which may supplement exercise programs. |
| Locations | A program map of current cancer specific exercise rehabilitation programs in Australia with contact details. |
| Resources | Includes generic templates of forms and templates which can be used by exercise professionals in their practice. |
