# Supplementary material for: A Cancer Exercise Toolkit Developed Using Co-Design: Mixed Methods Study
Source: JMIR Cancer. 2022 Apr 21;8(2):e34903. doi: 10.2196/34903 (PMC9073617; doi:10.2196/34903)
Supplement: Multimedia Appendix 6 [file cancer_v8i2e34903_app6.docx]

Appendix 6. Workshop 4 – Key website changes

| **Area** | **Description of changes** |
| --- | --- |
| General | - URL and website title changed to Cancer Exercise Toolkit - Logo created - Descriptor on home page describing to use Google Chrome for best functionality - Addition of “Contact us” section at bottom of home page |
| Navigation | - Subpages visible in top menu options - Sidebar added for specific website sections e.g. exercise prescription - Creation of separate tab for resources - Consistency of pop-out pages – those internal to website open within site and those external to website open in new browser |
| Content | - Addition of ‘Special Cancer Populations’ section including exercise for specific cancer types and age groups - Addition of further detail about cancer treatment and its impact on exercise assessment and prescription. Additional pages created for each treatment type e.g. Chemotherapy, Radiotherapy - Addition of precautions related to surgical procedures in precautions table - Addition of “Resources” page where all printable resources can be located together |
