# Supplementary figures and images for: A Cancer Exercise Toolkit Developed Using Co-Design: Mixed Methods Study
Source: JMIR Cancer. 2022 Apr 21;8(2):e34903. doi: 10.2196/34903 (PMC9073617; doi:10.2196/34903)

Appendix 7. Screenshots of Cancer Exercise toolkit

1. Homepage


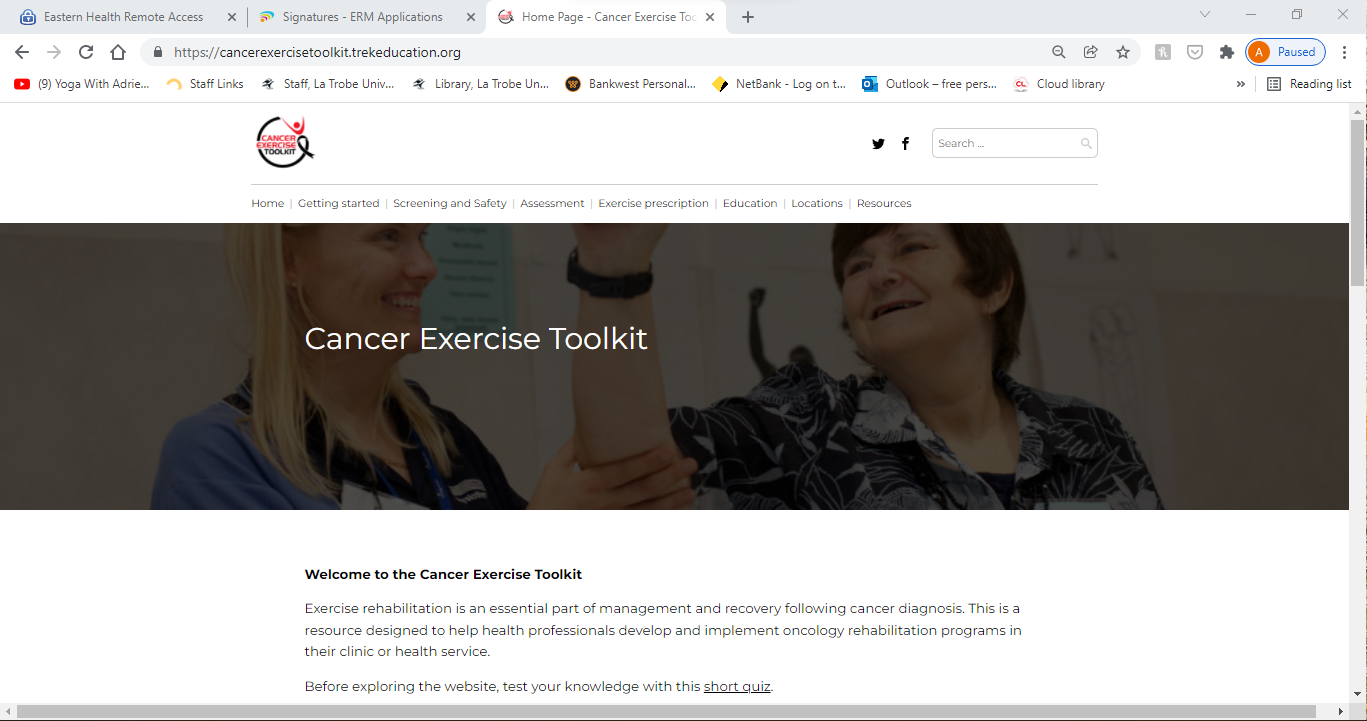

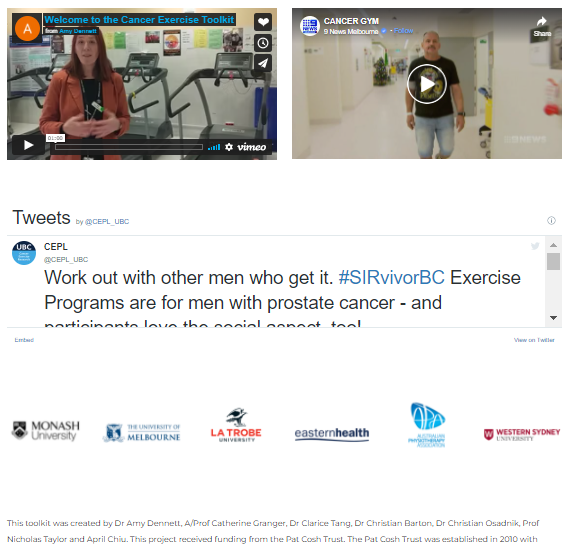


1. Patient education page


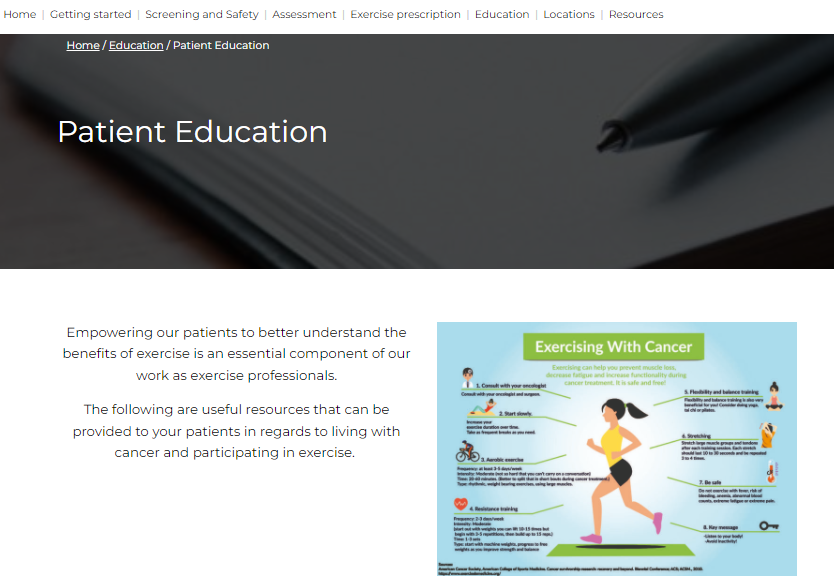

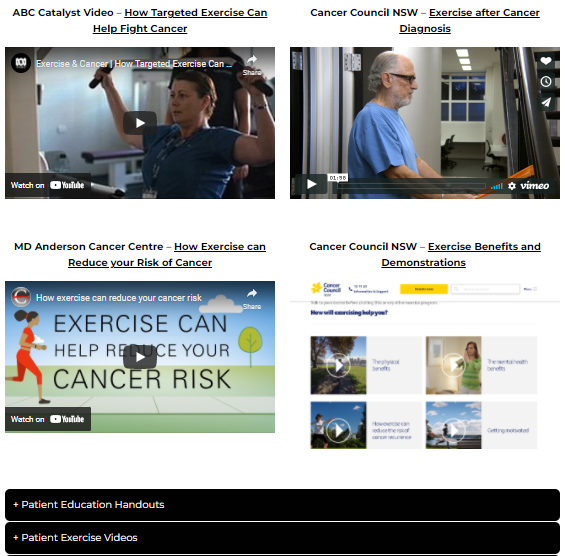

Supplement: Multimedia Appendix 7 [file cancer_v8i2e34903_app7.docx]
