# Supplementary material for: A Cancer Exercise Toolkit Developed Using Co-Design: Mixed Methods Study
Source: JMIR Cancer. 2022 Apr 21;8(2):e34903. doi: 10.2196/34903 (PMC9073617; doi:10.2196/34903)
Supplement: Multimedia Appendix 8 [file cancer_v8i2e34903_app8.docx]

Appendix 8. Cancer Exercise Toolkit visits

| **Page** | **Page views** |
| --- | --- |
| Homepage | 1472 |
| Getting started | 48 |
| Background | 124 |
| Managing referrals | 57 |
| Practical considerations | 57 |
| Forms and templates | 74 |
| Screening and safety | 72 |
| Precautions and contraindications | 199 |
| Red Flags | 98 |
| Chemotherapy | 63 |
| Radiotherapy | 38 |
| Immunotherapy | 44 |
| Targeted Therapy | 41 |
| Surgery | 32 |
| Bony metastases | 97 |
| Fatigue | 93 |
| Lymphodema | 62 |
| Special Cancer Populations | 35 |
| Breast | 22 |
| Prostate | 25 |
| Head and neck | 11 |
| Lung | 24 |
| Blood | 20 |
| Palliative care and advanced cancer | 62 |
| Paediatrics and young adults | 13 |
| Geriatric Oncology | 10 |
| Assessment | 52 |
| Subjective assessment | 123 |
| Objective assessment | 158 |
| Multidisciplinary screening | 56 |
| Reassessment and follow-up | 40 |
| Exercise prescription | 45 |
| Setting exercise prescription - Aerobic | 126 |
| Setting exercise prescription - Strength | 104 |
| Exercise modification and progression | 122 |
| Case studies | 99 |
| Education | 7 |
| Patient education | 208 |
| Clinician education | 190 |
| Multidisciplinary education | 54 |
| Locations^a^ | 209 |
| Resources | 183 |

**^a^**Includes any interaction with a specific program on the locations page
