# Supplementary material for: A Cancer Exercise Toolkit Developed Using Co-Design: Mixed Methods Study
Source: JMIR Cancer. 2022 Apr 21;8(2):e34903. doi: 10.2196/34903 (PMC9073617; doi:10.2196/34903)
Supplement: Multimedia Appendix 9 [file cancer_v8i2e34903_app9.docx]

Appendix 9. Detailed data related to Determinants of Implementation Behavior Questionnaire

1. Determinants of Implementation Behaviour Questionnaire reported as frequency on 1-7 likert scale

|  | **Strongly disagree**  **1** | | | **2** | **3** | | | **4** | **5** | **6** | | **Strongly**  **agree**  **7** |
| --- | --- | --- | --- | --- | --- | --- | --- | --- | --- | --- | --- | --- |
| 1. I know how to deliver Exercise Oncology Rehabilitation following the guidelines. | | | | | | | | | | | | |
| Pre-website | 21 (7) | | | 28 (9) | 30 (9) | | | 57 (18) | 63 (20) | 44 (14) | | 30 (9) |
| Post-website | 0 (0) | | | 2 (4) | 1 (2) | | | 5 (9) | 19 (35) | 15 (27) | | 13 (24) |
| 1. Objectives of Exercise Oncology Rehabilitation and my role in this are clearly defined for me. | | | | | | | | | | | | |
| Pre-website | 16 (5) | | | 38 (12) | 36 (11) | | | 59 (18) | 53 (17) | 34 (11) | | 37 (12) |
| Post-website | 1 (2) | | | 2 (4) | 2 (4) | | | 6 (11) | 17 (31) | 16 (29) | | 11 (20) |
| 1. With regard to Exercise Oncology Rehabilitation, I know what my responsibilities are. | | | | | | | | | | | | |
| Pre-website | 15 (5) | | | 30 (9) | 31 (10) | | | 52 (16) | 66 (21) | 47 (15) | | 32 (10) |
| Post-website | 1 (2) | | | 1 (2) | 1 (2) | | | 6 (11) | 13 (24) | 21 (38) | | 12 (22) |
| 1. In my work with Exercise Oncology Rehabilitation, I know exactly what is expected from me. | | | | | | | | | | | | |
| Pre-website | | 21 (7) | 33 (10) | | | 35 (11) | 63 (20) | | 58 (18) | | 39 (12) | 23 (7) |
| Post-website | | 2 (4) | 1 (2) | | | 1 (2) | 6 (11) | | 16 (29) | | 19 (35) | 10 (18) |
| 1. I have been trained in delivering Exercise Oncology Rehabilitation following the guidelines. | | | | | | | | | | | | |
| Pre-website | 69 (22) | | | 40 (13) | 23 (7) | | | 48 (15) | 34 (11) | 35 (11) | | 22 (7) |
| Post-website | 2 (4) | | | 3 (6) | 4 (7) | | | 9 (16) | 11 (20) | 16 (29) | | 10 (18) |
| 1. I have the skills to deliver Exercise Oncology Rehabilitation following the guidelines | | | | | | | | | | | | |
| Pre-website | 26 (8) | | | 17 (5) | 30 (9) | | | 43 (13) | 61 (19) | 53 (17) | | 40 (13) |
| Post-website | 0 (0) | | | 1 (2) | 3 (6) | | | 4 (7) | 17 (31) | 18 (33) | | 1. (22) |
| 1. I am practiced to deliver Exercise Oncology Rehabilitation following the guidelines. | | | | | | | | | | | | |
| Pre-website | 48 (15) | | | 31 (10) | 31 (10) | | | 51 (16) | 47 (15) | 33 (10) | | 29 (9) |
| Post-website | 1 (2) | | | 2 (4) | 3 (6) | | | 8 (15) | 13 (25) | 17 (31) | | 11 (20) |
| 1. I am confident that I can deliver Exercise Oncology Rehabilitation following the guidelines. | | | | | | | | | | | | |
| Pre-website | 23 (7) | | | 29 (9) | 32 (10) | | | 46 (14) | 58 (18) | 43 (13) | | 39 (12) |
| Post-website | 0 (0) | | | 3 (6) | 1 (2) | | | 6 (11) | 19 (35) | 11 (20) | | 15 (27) |
| 1. I am confident that I can deliver Exercise Oncology Rehabilitation following the guidelines even when other professionals with whom I deliver Exercise Oncology Rehabilitation do not do this. | | | | | | | | | | | | |
| Pre-website | 24 (8) | | | 27 (8) | 42 (13) | | | 45 (14) | 58 (18) | 40 (13) | | 33 (10) |
| Post-website | 1 (2) | | | 2 (4) | 1 (2) | | | 8 (15) | 17 (31) | 15 (27) | | 11 (20) |
| 1. I am confident that I can deliver Exercise Oncology Rehabilitation following the guidelines even when there is little time | | | | | | | | | | | | |
| Pre-website | 27 (8) | | | 28 (9) | 55 (17) | | | 53 (17) | 55 (17) | 34 (11) | | 16 (5) |
| Post-website | 1 (2) | | | 4 (7) | 3 (6) | | | 12 (22) | 18 (33) | 11 (20) | | 6 (11) |
| 1. I am confident that I can deliver Exercise Oncology Rehabilitation following the guidelines even when participants are not motivated. | | | | | | | | | | | | |
| Pre-website | 27 (8) | | | 36 (11) | 53 (17) | | | 52 (16) | 70 (22) | 16 (5) | | 14 (4) |
| Post-website | 0 (0) | | | 5 (9) | 3 (6) | | | 11 (20) | 19 (35) | 14 (26) | | 3 (6) |
| 1. I have control over delivering Exercise Oncology Rehabilitation following the guidelines. | | | | | | | | | | | | |
| Pre-website | 24 (8) | | | 29 (9) | 31 (10) | | | 63 (20) | 53 (17) | 42 (13) | | 26 (8) |
| Post-website | 1 (2) | | | 3 (6) | 3 (6) | | | 11 (20) | 15 (27) | 13 (24) | | 9 (16) |
| 23. If I deliver Exercise Oncology Rehabilitation following the guidelines Exercise Oncology Rehabilitation will be most effective**the time** | | | | | | | | | | | | |
| Pre-website | 2 (1) | | | 0 (0) | 6 (2) | | | 20 (6) | 46 (14) | 79 (25) | | 72 (23) |
| Post-website | 0 (0) | | | 0 (0) | 1 (2) | | | 2 (4) | 6 (11) | 20 (36) | | 19 (35) |
| 24. If I deliver Exercise Oncology Rehabilitation following the guidelines, participants will appreciate this. | | | | | | | | | | | | |
| Pre-website | 1 (0.3) | | | 0 (0) | 6 (2) | | | 44 (14) | 53 (17) | 66 (21) | | 54 (17) |
| Post-website | 0 (0) | | | 0 (0) | 2 (4) | | | 4 (7) | 9 (16) | 17 (31) | | 16 (29) |
| 25. If I deliver Exercise Oncology Rehabilitation following the guidelines, this will strengthen the collaboration with professionals with whom I deliver Exercise Oncology Rehabilitation. | | | | | | | | | | | | |
| Pre-website | 2 (1) | | | 1 (0.3) | 9 (3) | | | 23 (7) | 45 (14) | 78 (24) | | 66 (21) |
| Post-website | 0 (0) | | | 0 (0) | 1 (2) | | | 3 (6) | 11 (20) | 14 (26) | | 19 (35) |
| 26. If I deliver Exercise Oncology Rehabilitation following the Guidelines, I will feel satisfied. | | | | | | | | | | | | |
| Pre-website | 1 (0.3) | | | 0 (0) | 5 (2) | | | 18 (6) | 48 (15) | 73 (23) | | 79 (25) |
| Post-website | 0 (0) | | | 1 (2) | 1 (2) | | | 2 (4) | 5 (9) | 20 (36) | | 19 (35) |
| 27. If I deliver Exercise Oncology Rehabilitation following the Guidelines, it will help participants to be more physically active. | | | | | | | | | | | | |
| Pre-website | 1 (0.3) | | | 0 (0) | 8 (3) | | | 15 (5) | 33 (10) | 87 (27) | | 80 (25) |
| Post-website | 0 (0) | | | 0 (0) | 1 (2) | | | 5 (33) | 6 (33) | 18 (33) | | 18 (33) |
| 28. It is possible to tailor Exercise Oncology Rehabilitation to participants’ needs. | | | | | | | | | | | | |
| Pre-website | 1 (0.3) | | | 0 (0) | 6 (2) | | | 19 (6) | 40 (13) | 64 (20) | | 94 (29) |
| Post-website | 0 (0) | | | 0 (0) | 1 (2) | | | 3 (6) | 6 (11) | 17 (31) | | 21 (38) |
| 29. It is possible to tailor Exercise Oncology Rehabilitation to professionals’ needs. | | | | | | | | | | | | |
| Pre-website | 2 (1) | | | 0 (0) | 10 (3) | | | 42 (13) | 63 (20) | 60 (19) | | 46 (14) |
| Post-website | 0 (0) | | | 0 (0) | 1 (2) | | | 7 (13) | 15 (27) | 16 (29) | | 9 (16) |
| 30. Exercise Oncology Rehabilitation costs little time to deliver. | | | | | | | | | | | | |
| Pre-website | 7 (2) | | | 19 (6) | 34 (11) | | | 78 (24) | 44 (14) | 29 (9) | | 12 (4) |
| Post-website | 2 (4) | | | 3 (6) | 6 (11) | | | 14 (26) | 15 (27) | 5 (9) | | 3 (6) |
| 31. Exercise Oncology Rehabilitation is compatible with daily practice | | | | | | | | | | | | |
| Pre-website | 1 (0.3) | | | 4 (1) | 23 (7) | | | 51 (16) | 53 (17) | 58 (18) | | 32 (10) |
| Post-website | 0 (0) | | | 1 (2) | 1 (2) | | | 6 (11) | 19 (35) | 13 (24) | | 8 (15) |
| 32. Exercise Oncology Rehabilitation is simple to deliver | | | | | | | | | | | | |
| Pre-website | 6 (2) | | | 9 (3) | 34 (11) | | | 78 (24) | 51 (16) | 29 (9) | | 15 (5) |
| Post-website | 0 (0) | | | 3 (6) | 3 (6) | | | 15 (27) | 12 (22) | 12 (22) | | 3 (6) |
| 33. Most people who are important to me think that I should deliver Exercise Oncology Rehabilitation following the guidelines. | | | | | | | | | | | | |
| Pre-website | 5 (2) | | | 8 (3) | 16 (5) | | | 63 (20) | 48 (15) | 46 (14) | | 36 (11) |
| Post-website | 0 (0) | | | 1 (2) | 1 (2) | | | 8 (15) | 14 (26) | 15 (27) | | 9 (16) |
| 34. Professionals with whom I deliver Exercise Oncology Rehabilitation think I should deliver Exercise Oncology Rehabilitation following the guidelines. | | | | | | | | | | | | |
| Pre-website | 4 (1) | | | 12 (4) | 13 (4) | | | 51 (16) | 45 (14) | 55 (17) | | 41 (13) |
| Post-website | 0 (0) | | | 1 (2) | 2 (4) | | | 6 (11) | 11 (20) | 13 (24) | | 15 (27) |
| 35. Professionals with whom I deliver Exercise Oncology Rehabilitation deliver Exercise Oncology Rehabilitation following the guidelines | | | | | | | | | | | | |
| Pre-website | 8 (3) | | | 15 (5) | 20 (6) | | | 59 (18) | 51 (16) | 40 (13) | | 27 (8) |
| Post-website | 0 (0) | | | 3 (6) | 0 (0) | | | 10 (18) | 15 (27) | 12 (22) | | 7 (13) |
| 36. Other professionals who work with Exercise Oncology Rehabilitation deliver Exercise Oncology Rehabilitation following the guidelines | | | | | | | | | | | | |
| Pre-website | 3 (1) | | | 18 (6) | 30 (9) | | | 72 (23) | 46 (14) | 30 (9) | | 21 (7) |
| Post-website | 1 (2) | | | 3 (6) | 1 (2) | | | 12 (22) | 13 (24) | 10 (18) | | 7 (13) |
| 37. I can count on support from professionals with whom I deliver Exercise Oncology Rehabilitation when things get tough around delivering Exercise Oncology Rehabilitation following the guidelines. | | | | | | | | | | | | |
| Pre-website | 4 (1) | | | 8 (3) | 25 (8) | | | 58 (18) | 64 (20) | 40 (13) | | 21 (7) |
| Post-website | 1 (2) | | | 3 (6) | 0 (0) | | | 13 (24) | 14 (26) | 12 (22) | | 4 (7) |
| 38. Professionals with whom I deliver Exercise Oncology Rehabilitation are willing to listen to my problems with delivering Exercise Oncology Rehabilitation following the guidelines. | | | | | | | | | | | | |
| Pre-website | 7 (2) | | | 7 (2) | 18 (6) | | | 71 (22) | 55 (17) | 38 (12) | | 24 (8) |
| Post-website | 1 (2) | | | 2 (4) | 1 (2) | | | 11 (20) | 16 (29) | 12 (22) | | 4 (7) |
| 39. Professionals with whom I deliver Exercise Oncology Rehabilitation are helpful with delivering Exercise Oncology Rehabilitation following the guidelines. | | | | | | | | | | | | |
| Pre-website | 7 (2) | | | 11 (3) | 21 (7) | | | 73 (23) | 58 (18) | 34 (11) | | 16 (5) |
| Post-website | 1 (2) | | | 2 (4) | 2 (4) | | | 12 (22) | 15 (27) | 10 (18) | | 5 (9) |
| 40. Delivering Exercise Oncology Rehabilitation following the guidelines is something I do automatically. | | | | | | | | | | | | |
| Pre-website | 16 (5) | | | 23 (7) | 24 (8) | | | 43 (13) | 61 (19) | 41 (13) | | 11 (3) |
| Post-website | 1 (2) | | | 2 (4) | 0 (0) | | | 6 (11) | 21 (38) | 9 (16) | | 8 (15) |
| 41. Delivering Exercise Oncology Rehabilitation following the guidelines is something I do without having to consciously remember | | | | | | | | | | | | |
| Pre-website | 18 (6) | | | 26 (8) | 30 (9) | | | 41 (13) | 57 (18) | 37 (12) | | 10 (3) |
| Post-website | 2 (4) | | | 2 (4) | 2 (4) | | | 5 (9) | 21 (38) | 11 (20) | | 4 (7) |
| 42. Delivering Exercise Oncology Rehabilitation following the guidelines is something I do without thinking. | | | | | | | | | | | | |
| Pre-website | 19 (6) | | | 33 (10) | 33 (10) | | | 43 (13) | 52 (16) | 31 (10) | | 8 (3) |
| Post-website | 2 (4) | | | 2 (4) | 2 (4) | | | 10 (18) | 17 (31) | 7 (13) | | 7 (13) |
| 43. Delivering Exercise Oncology Rehabilitation following the guidelines is something I start doing before I realize I am doing it | | | | | | | | | | | | |
| Pre-website | 18 (6) | | | 25 (8) | 29 (9) | | | 45 (14) | 56 (18) | 33 (10) | | 12 (4) |
| Post-website | 2 (4) | | | 2 (4) | 2 (4) | | | 6 (11) | 17 (31) | 10 (18) | | 8 (15) |
| 44. Delivering Exercise Oncology Rehabilitation following the guidelines is something I seldom forget. | | | | | | | | | | | | |
| Pre-website | 23 (7) | | | 23 (7) | 19 (6) | | | 48 (15) | 58 (18) | 29 (9) | | 19 (6) |
| Post-website | 3 (6) | | | 2 (4) | 1 (2) | | | 7 (13) | 19 (35) | 10 (18) | | 5 (9) |
| 45. Delivering Exercise Oncology Rehabilitation following the guidelines is something I often forget. | | | | | | | | | | | | |
| Pre-website | 45 (14) | | | 65 (20) | 39 (12) | | | 37 (12) | 18 (6) | 11 (3) | | 4 (1) |
| Post-website | 14 (26) | | | 17 (31) | 5 (9) | | | 4 (7) | 5 (9) | 1 (2) | | 1 (2) |
|  | **Not Very Worthwhile** | | |  |  | | |  |  |  | | **Very worthwhile** |
| 20. For me, delivering Exercise Oncology Rehabilitation following the guidelines is | | | | | | | | | | | | |
| Pre-website | 0 (0) | | | 3 (1) | 14 (4) | | | 44 (14) | 48 (15) | 53 (17) | | 88 (28) |
| Post-website | 0 (0) | | | 0 (0) | 3 (6) | | | 5 (9) | 6 (11) | 16 (29) | | 23 (42) |
|  | **Not pleasurable at all** | | |  |  | | |  |  |  | | **Very pleasurable** |
| 21. For me, delivering Exercise Oncology Rehabilitation following the guidelines is | | | | | | | | | | | |  |
| Pre-website | 2 (1) | | | 3 (1) | 18 (6) | | | 48 (15) | 65 (20) | 51 (16) | | 55 (17) |
| Post-website | 0 (0) | | | 1 (2) | 1 (2) | | | 5 (9) | 16 (29) | 12 (22) | | 17 (31) |
|  | **Not interesting at all** | | |  |  | | |  |  |  | | **Very interesting** |
| 22. For me, delivering Exercise Oncology Rehabilitation following the guidelines is | | | | | | | | | | | | |
| Pre-website | 0 (0) | | | 2 (1) | 15 (5) | | | 37 (12) | 65 (20) | 49 (15) | | 74 (23) |
| Post-website | 0 (0) | | | 1 | 0 (0) | | | 6 (11) | 12 (22) | 13 (24) | | 20 (36) |
|  | **Very Difficult** | | |  |  | | |  |  |  | | **Very Easy** |
| 13. For me, delivering Exercise Oncology Rehabilitation following the guidelines is | | | | | | | | | | | | |
| Pre-website | 11 (3) | | | 18 (6) | 41 (13) | | | 74 (23) | 70 (22) | 30 (9) | | 10 (3) |
| Post-website | 0 (0) | | | 2 (4) | 1 (2) | | | 12 (22) | 19 (35) | 15 (27) | | 4 (7) |
| 14. For me, performing the intake is | | | | | | | | | | | | |
| Pre-website | 19 (6) | | | 31 (10) | 46 (14) | | | 67 (21) | 59 (18) | 22 (7) | | 7 (2) |
| Post-website | 2 (4) | | | 4 (7) | 3 (6) | | | 16 (29) | 12 (22) | 12 (22) | | 4 (7) |
| 15. For me, delivering the training program is | | | | | | | | | | | | |
| Pre-website | 18 (6) | | | 23 (7) | 46 (14) | | | 55 (17) | 65 (20) | 35 (11) | | 9 (3) |
| Post-website | 1 (2) | | | 2 (4) | 4 (7) | | | 14 (26) | 12 (22) | 14 (26) | | 6 (11) |
| 16. For me, performing the evaluation is | | | | | | | | | | | | |
| Pre-website | 16 (5) | | | 25 (8) | 46 (14) | | | 64 (20) | 59 (18) | 31 (10) | | 10 (3) |
| Post-website | 2 (4) | | | 3 (6) | 4 (7) | | | 14 (26) | 15 (27) | 12 (22) | | 3 (6) |
| 17. For me, giving attention to participant’s maintenance of physical activity behavior outside Exercise Oncology Rehabilitation is | | | | | | | | | | | | |
| Pre-website | 14 (4) | | | 31 (10) | 40 (13) | | | 61 (19) | 60 (19) | 31 (10) | | 13 (4) |
| Post-website | 3 (6) | | | 2 (4) | 4 (7) | | | 10 (18) | 21 (38) | 11 (20) | | 2 (4) |
| 18. For me, reporting about Exercise Oncology Rehabilitation to the referring professional is | | | | | | | | | | | | |
| Pre-website | 13 (4) | | | 25 (8) | 46 (14) | | | 56 (18) | 54 (17) | 41 (13) | | 15 (5) |
| Post-website | 2 (4) | | | 4 (7) | 4 (7) | | | 13 (24) | 14 (26) | 11 (20) | | 5 (9) |
|  | **Not useful at all** | | |  |  | | |  |  |  | | **Very useful** |
| 19. For me, delivering Exercise Oncology Rehabilitation following the guidelines is | | | | | | | | | | | | |
| Pre-website | 1 (0.3) | | | 1 (0.3) | 22 (7) | | | 44 (14) | 66 (21) | 53 (17) | | 62 (19) |
| Post-website | 0 (0) | | | 0 (0) | 3 (6) | | | 5 (9) | 7 (13) | 19 (35) | | 19 (35) |

Baseline n=273, 3-month follow-up N=55

Percentages may not equal 100% due to missing data

b) Determinants of Implementation Behaviour Questionnaire reported as median and IQR

| Question | Baseline  Median (IQR)  n=273 | Follow-up  Median (IQR)  n=55 | Between group difference p= |
| --- | --- | --- | --- |
| 1. I know how to deliver Exercise Oncology Rehabilitation following the guidelines. | 5 (3-6) | 6 (5-6) | **<0.001** |
| 1. Objectives of Exercise Oncology Rehabilitation and my role in this are clearly defined for me. | 4 (3-6) | 5 (5-6) | **<0.001** |
| 1. With regard to Exercise Oncology Rehabilitation, I know what my responsibilities are. | 5 (3-6) | 6 (5-6) | **<0.001** |
| 1. In my work with Exercise Oncology Rehabilitation, I know exactly what is expected from me. | 4 (3-5) | 6 (5-6) | **<0.001** |
| 1. I have been trained in delivering Exercise Oncology Rehabilitation following the guidelines. | 4 (1-5) | 6 (4-6) | **<0.001** |
| 1. I have the skills to deliver Exercise Oncology Rehabilitation following the guidelines. | 5 (3-6) | 6 (5-6) | **<0.001** |
| 1. I am practiced to deliver Exercise Oncology Rehabilitation following the guidelines. | 4 (2-5) | 6 (4-6) | **<0.001** |
| 1. I am confident that I can deliver Exercise Oncology Rehabilitation following the guidelines. | 5 (3-6) | 5 (5-7) | **<0.001** |
| 1. I am confident that I can deliver Exercise Oncology Rehabilitation following the guidelines even when other professionals with whom I deliver Exercise Oncology Rehabilitation do not do this. | 4 (3-6) | 5 (5-6) | **<0.001** |
| 1. I am confident that I can deliver Exercise Oncology Rehabilitation following the guidelines even when there is little time. | 4 (3-5) | 5 (4-6) | <0.001 |
| 1. I am confident that I can deliver Exercise Oncology Rehabilitation following the guidelines even when participants are not motivated. | 4 (3-5) | 5 (4-6) | **<0.001** |
| 1. I have control over delivering Exercise Oncology Rehabilitation following the guidelines. | 4 (3-6) | 5 (4-6) | 0.001 |
| 1. For me, delivering Exercise Oncology Rehabilitation following the guidelines is   (very difficult – very easy) | 4 (3-5) | 5 (4-6) | <0.001 |
| 1. For me, performing the intake is (very difficult – very easy) | 4 (3-5) | 5 (4-6) | 0.001 |
| 1. For me, delivering the training program is (very difficult – very easy). | 4 (3-5) | 5 (4-6) | <0.001 |
| 1. For me, performing the evaluation is (very difficult – very easy) | 4 (3-5) | 5 (4-6) | 0.008 |
| 1. For me, giving attention to participant’s maintenance of physical activity behavior outside Exercise Oncology Rehabilitation is (very difficult – very easy) | 4 (3-5) | 5 (4-5.5) | 0.009 |
| 1. For me, reporting about the Exercise Oncology Rehabilitation to the referring professional is   (very difficult – very easy) | 4 (3-5) | 5 (4-6) | 0.053 |
| 1. For me, delivering Exercise Oncology Rehabilitation following the guidelines is   (not useful at all – very useful) | 5 (4-6.5) | 6 (5-7) | 0.005 |
| 1. For me, delivering Exercise Oncology Rehabilitation following the guidelines is   (not worthwhile at all – very worthwhile) | 6 (5-7) | 6 (5-7) | 0.069 |
| 1. For me, delivering Exercise Oncology Rehabilitation following the guidelines is   (not pleasurable at all – very pleasurable) | 5 (4-6) | 6 (5-7) | 0.027 |
| 1. For me, delivering Exercise Oncology Rehabilitation following the guidelines is   (not interesting at all – very interesting) | 6 (5-7) | 6 (5-7) | 0.086 |
| 1. If I deliver Exercise Oncology Rehabilitation following the guidelines Exercise Oncology Rehabilitation will be most effective. | 6 (5-7) | 6 (6-7) | 0.088 |
| 1. If I deliver Exercise Oncology Rehabilitation following the guidelines, participants will appreciate this. | 6 (5-6) | 6 (5-7) | 0.053 |
| 1. If I deliver Exercise Oncology Rehabilitation following the guidelines, this will strengthen the collaboration with professionals with whom I deliver Exercise Oncology Rehabilitation. | 6 (5-7) | 6 (5-7) | 0.185 |
| 1. If I deliver Exercise Oncology Rehabilitation following the Guidelines, I will feel satisfied. | 6 (5-7) | 6 (6-7) | 0.223 |
| 1. If I deliver Exercise Oncology Rehabilitation following the Guidelines, it will help participants to be more physically active. | 6 (5-7) | 6 (5.3-7) | 0.872 |
| 1. It is possible to tailor Exercise Oncology Rehabilitation to participants’ needs? | 6 (5-7) | 6 (6-7) | 0.471 |
| 1. It is possible to tailor Exercise Oncology Rehabilitation to professionals’ needs? | 5 (5-6) | 6 (5-6) | 0.502 |
| 1. Exercise Oncology Rehabilitation costs little time to deliver. | 4 (3-5) | 4 (4-5) | 0.425 |
| 1. Exercise Oncology Rehabilitation is compatible with daily practice | 5 (4-6) | 5 (5-6) | 0.113 |
| 1. Exercise Oncology Rehabilitation is simple to deliver | 4 (4-5) | 5 (4-6) | 0.058 |
| 1. Most people who are important to me think that I should deliver Exercise Oncology Rehabilitation following the guidelines. | 5 (4-6) | 5.5 (5-6) | 0.023 |
| 1. Professionals with whom I deliver Exercise Oncology Rehabilitation think I should deliver Exercise Oncology Rehabilitation following the guidelines. | 5 (4-6) | 6 (5-7) | 0.012 |
| 1. Professionals with whom I deliver Exercise Oncology Rehabilitation deliver Exercise Oncology Rehabilitation following the guidelines | 5 (4-6) | 5 (4-6) | 0.028 |
| 1. Other professionals who work with Exercise Oncology Rehabilitation deliver Exercise Oncology Rehabilitation following the guidelines | 4 (4-5) | 5 (4-6) | 0.155 |
| 1. I can count on support from professionals with whom I deliver Exercise Oncology Rehabilitation when things get tough around delivering Exercise Oncology Rehabilitation following the guidelines. | 5 (4-6) | 5 (4-6) | 0.063 |
| 1. Professionals with whom I deliver Exercise Oncology Rehabilitation are willing to listen to my problems with delivering Exercise Oncology Rehabilitation following the guidelines. | 5 (4-6) | 5 (4-6) | 0.155 |
| 1. Professionals with whom I deliver Exercise Oncology Rehabilitation are helpful with delivering Exercise Oncology Rehabilitation following the guidelines. | 4 (4-5) | 5 (4-6) | 0.063 |
| 1. Delivering Exercise Oncology Rehabilitation following the guidelines is something I do automatically. | 5 (3-5) | 5 (5-6) | <0.001 |
| 1. Delivering Exercise Oncology Rehabilitation following the guidelines is something I do without having to consciously remember | 4 (3-5) | 5 (5-6) | 0.001 |
| 1. Delivering Exercise Oncology Rehabilitation following the guidelines is something I do without thinking. | 4 (3-5) | 5 (4-6) | 0.001 |
| 1. Delivering Exercise Oncology Rehabilitation following the guidelines is something I start doing before I realize I am doing it | 4 (3-5) | 5 (4-6) | <0.001 |
| 1. Delivering Exercise Oncology Rehabilitation following the guidelines is something I seldom forget. | 4 (3-5) | 5 (4-6) | 0.008 |
| 1. Delivering Exercise Oncology Rehabilitation following the guidelines is something I often forget.^a^ | 2 (2-4) | 2 (1-3) | 0.088 |

^a^Reverse worded item, lower score is better

^b^Significance level set to 0.001 (Bonferroni adjustment)

^c^Bold indicates significance after sensitivity analysis applied

^d^n=3 responses excluded at follow-up as participants indicated they never accessed the website
